# Identification and caste-dependent expression patterns of DNA methylation associated genes in *Bombus terrestris*

**DOI:** 10.1038/s41598-018-20831-1

**Published:** 2018-02-05

**Authors:** Beibei Li, Li Hou, Dan Zhu, Xilian Xu, Shiheng An, Xianhui Wang

**Affiliations:** 1 College of Plant Protection, Henan Agricultural University, Zhengzhou, 450002 China; 2 Institute of Zoology, Chinese Academy of Sciences, Beijing, 100101 China; 3 Institute of Plant and Environment Protection, Beijing Academy of Agriculture and Forestry Sciences, Beijing, 100097 China

## Abstract

DNA methylation has been proposed to play critical roles in caste fate and behavioral plasticity in bumblebees, however, there is little information on its regulatory mechanisms. Here, we identified six important genes mediating the modification of DNA methylation and determined their expression patterns in the bumblebee *Bombus terrestris*. There is a complete functional DNA methylation system, including four DNA methyltransferases (DNMT1a, DNMT1b, DNMT2, and DNMT3), a DNA demethylase (Ten-eleven translocation), and a methyl-CpG-binding domain protein in *B. terrestris*. Most of these genes were highly expressed in fat bodies and gonads but lowly expressed in antennae and brains of bumblebee adults. Besides, these genes exhibited caste-specific expression patterns in bumblebees, with higher transcription levels in queens than workers and drones. Whereas their expression levels showed no remarkable difference in queenright and queenless workers. These results suggested potential roles of DNA methylation-related genes in caste differentiation in bumblebees.

## Introduction

The bumblebee *Bombus terrestris* is one of the most effective plant pollinators featuring important economic and ecological values^1^. *B. terrestris* exhibits striking morphological and behavioral plasticities involved in unique caste differentiation^2^. Their morphology, physiology, behavior, longevity, and other life-history traits dramatically differ between queens and workers although they are derived from the same genome^3^. Reproduction plasticity also occurs between workers depending on social context in bumblebees^4^. In general, workers are sterile in the colony, but at the end of the colony, some workers become fertile and lay unfertilized eggs^5^. Besides, after removal of the queen, workers develop ovaries rapidly and are able to lay male destined eggs as early as the fifth day after emergence^6^. A series of studies suggested that several factors, such as nutrition, juvenile hormone, and social environments, play important roles in caste differentiation in *B. terrestris*^7,8^. However, the molecular mechanisms underlying caste differentiation and worker reproductive plasticity remain largely uncharacterized.

DNA methylation is a prominent epigenetic modification that plays essential roles in gene imprinting, X-chromosome inactivation, gene silencing and other significant biological processes^9,10^. Methylcytosine, a methyl group from S-adenosyl methionine is transferred to the fifth position on the pyrimidine group, which is one of the major DNA methylation modifications. Recently, DNA cytosine methylation has been suggested to be associated with caste differentiation in social insects^11,12^. After silencing the expression of the de novo methyltransferase *DNMT3*, the majority of worker-destined larvae emerged as queens with fully developed ovaries in the honeybee *Apis mellifera*^13^. Substantial differences in DNA methylation were identified between nurses and foragers, and methylation levels of a large number of genes showed great changes when reverting foragers back to nurses^14^. Over 500 different methylated genes have been identified in adult brains between queens and workers, and nearly all methylated cytosines are located in CpG dinucleotides in exons^15^. Another study indicated that there are 2399 genes showed significant differences of methylation in larval heads between queens and workers. Some of these genes are related to juvenile hormone and insulin pathways, which regulate caste determination in honeybees^16^. In addition, substantial differentially methylated genes were found between different castes in *Zootermopsis nevadensis*^17^ and *Camponotus floridanus*^18^.

Intricate cytosine methylation dynamics are achieved by methylation and demethylation processes catalyzed by methyltransferases and demethylases respectively. DNA methyltransferases (DNMTs) are divided into three types according to their functions. DNMT1 serves as maintenance methyltransferase, which is involved in the maintenance of cytosine methylation patterns during DNA replication^19^. DNMT3 is responsible for the establishment of DNA methylation during development, as the de novo methyltransferase^20^. DNMT2 also considered a methyltransferase because it features a conserved DNA methyltransferase catalytic domain. However, DNMT2 was proven to play an essential role in tRNA methylation^21^. Methyl-CpG-binding domain proteins (MBDs) can recognize and specifically bind to methylated sites, causing chromatin structural modification and remodeling by recruitment of repressive complexes, which are extremely imperative in DNA methylation mediated gene silencing^22^. Positive DNA demethylation mainly depends on the double oxygenase ten-eleven translocation protein (TET) through iterative oxidation reactions^23^.

Divergent levels of DNA methylation in *B. terrestris* have been reported at different developmental stages and genders^24^, and some DNA methylation-related genes have been mentioned in previous studies^25,26^, however, those genes involved in DNA methylation have not been characterised fully and the functional roles of these genes are not clear in the bumblebee. In this study, we characterized six key genes related to DNA methylation and determined their expression patterns in *B. terrestris* to lay the foundation for elucidating epigenetic mechanisms of caste differentiation in bumblebees.

## Results

### Sequence analysis of genes involved in DNA methylation from *B. terrestris*

Nucleotide sequences of six genes involved in DNA methylation in *B. terrestris* were obtained from the database of National Center for Biotechnology Information. Bioinformatically, we acquired four DNMTs (DNMT1a, DNMT1b, DNMT2, and DNMT3), a DNA demethylase (TET2), and a methyl-CpG-binding protein (MBD3). Supplementary Table S2 provides open reading frames (ORFs), numbers of amino acids, predicted relative molecular weights, and isoelectric points of these proteins.

**Figure 1:**
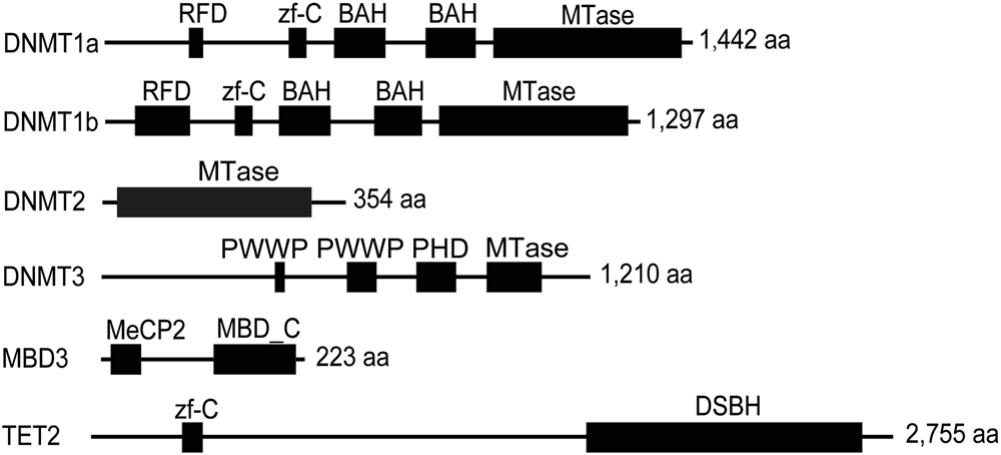
Predicted protein domains encoded by DNA methylation-related genes from *B. terrestris*. RFD: cytosine specific DNA methyltransferase replication foci domain; zf-C: CXXC zinc finger domain; BAH: bromo-adjacent homology domain; MTase: site-specific DNA-cytosine methylase domain; PWWP: domain with conserved PWWP motif; PHD: plant homeodomain; MeCP2: methyl-CpG-binding domain; MBD_C: C-terminal domain of methyl-CpG-binding protein; DSΒH: double-stranded beta helix fold domain.

By comparing domain structures of these genes, we found that DNMT1a and DNMT1b proteins had the same domain structures (Fig. 1). Both DNMT1s contain a replication foci (RFD) domain, a CXXC zinc finger (zf-C) domain, two bromo-adjacent homology (BAH) domains, and a site-specific DNA-cytosine methylase (MTase) domain. The MTase domain is responsible for cytosine methylation^27^. DNMT2 also contains a MTase domain (Fig. 1). Other than a MTase domain, two PWWP domains and a plant homeodomain (PHD) were also observed in DNMT3 (Fig. 1). The PHD domain serves as the catalytic center of DNMT3, which is essential for de novo methylation. MBD3 comprises a MeCP2 domain that can recognize methylated CpG dinucleotides specifically, and a MBD_C domain that is a typical characteristic of MBDs (Fig. 1). TET2 contains a zf-C domain and a double-stranded beta helix fold (DSΒH) domain (Fig. 1), the latter domain is involved in oxidizing reaction in DNA demethylation^28^. Above all, the bumblebee *B. terrestris* possesses a functional suite of DNA methylation-associated proteins, implying the possible important roles of DNA methylation in the bumblebee.

### Phylogenetic analysis of genes involved in DNA methylation from *B. terrestris* and other insects

The homologs of methylation-related genes in all insect species were firstly searched by using these sequences from the mice as seed sequences in NCBI database with a Blast tool. And then, we selected 11 insect species with the available whole genome sequences from six orders, including *A. mellifera*, *Anopheles gambiae*, *Acyrthosiphon pisum*, *Bombyx mori*, *B. terrestris*, *Camponotus floridanus*, *Drosophila melanogaster*, *Plutella xylostella*, *Tribolium castaneum*, *Nasonia vitripennis* and *Z. nevadensis* to constructed the phylogenetic trees. Nevertheless, some genes were lost in the genomes from several species or were not found based on the gene database of NCBI. Such as, *D. melanogaster* genome lacks DNMT1 and DNMT3^29^. Although DNMT1, DNMT2 and DNMT3 were reported in *A. pisum* genome from previous study^30^, we have not found the sequences of DNMT2 and DNMT3 of *A. pisum* according to the NCBI gene database. Thus, not all of DNA methylation-related genes from these species were included in phylogenetic analysis.

**Figure 2:**
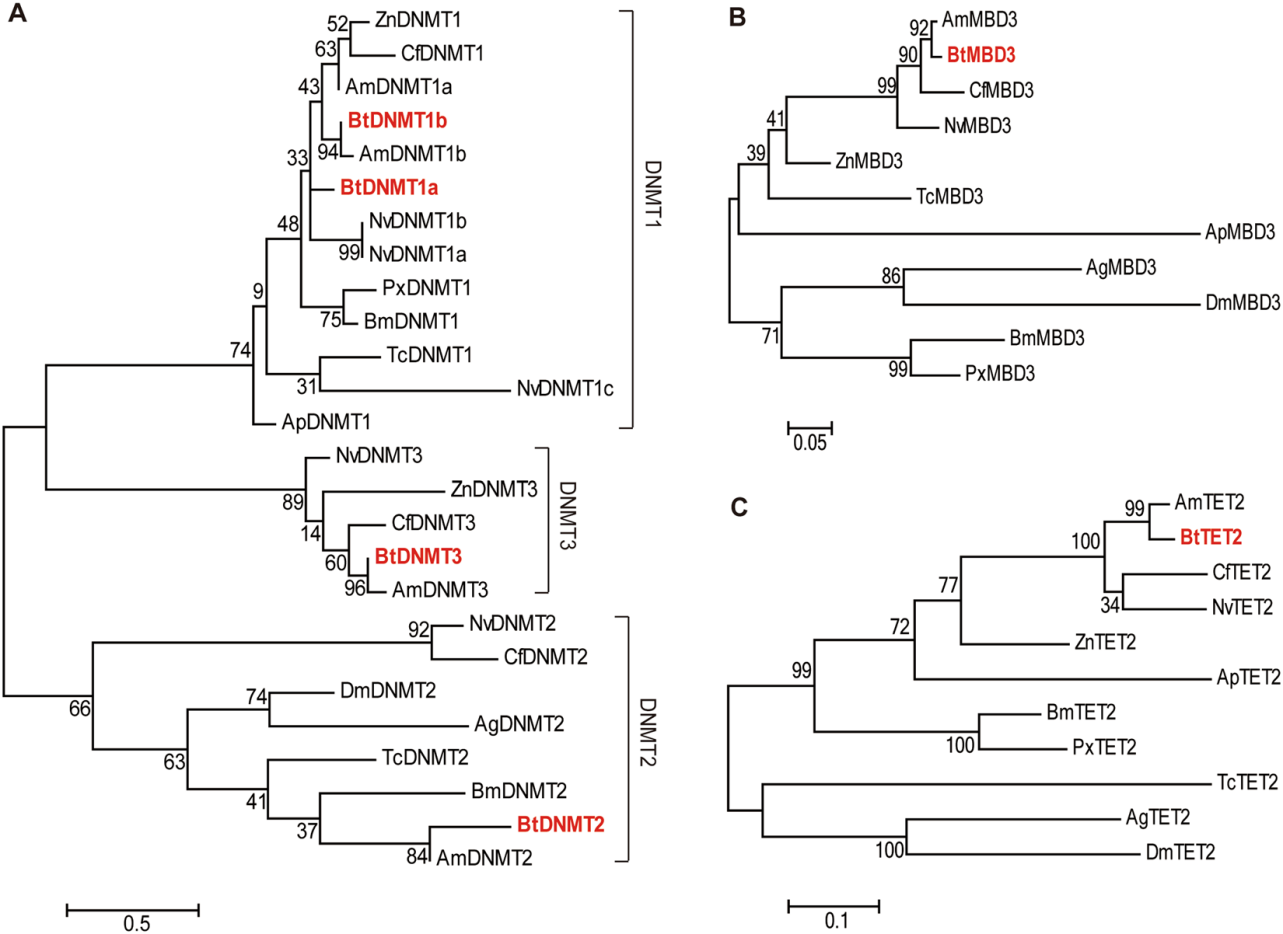
Maximum Likelihood-based phylogenetic trees of DNA methylation-related proteins. (**A**) Phylogenic tree based on amino acid sequences of DNMT1, DNMT2, and DNMT3 from *B. terrestris* and other insects. (**B**) Phylogenic tree based on amino acid sequences of MBD3 from *B. terrestris* and other insects. (**C**) Phylogenic tree based on amino acid sequences of TET2 from *B. terrestris* and other insects. Genes from *B. terrestris* are highlighted in bold red. Taxon abbreviations: Bt, *B. terrestris*; Am, *A. mellifera*; Ag, *A. gambiae*; Ap, *A. pisum*; Bm, *B. mori*; Cf, *C. floridanus*; Dm, *D. melanogaster*; Px, *P. xylostella*; Tc, *T. castaneum*; Nv, *N. vitripennis*; Zn, *Z. nevadensis*. Gene names, species names and GenBank accession numbers were shown in Supplementary Table S3.

DNMT1a, DNMT1b, DNMT2, and DNMT3 from different species were grouped to form a monophyletic clade respectively (Fig. 2A). Two members of DNMT1 from *B. terrestris* were clustered with the homologous proteins of other insects. The relationship of DNMT1a between *B. terrestris* and *A. mellifera* was closer. DNMT1b in *B. terrestris* showed close evolutionary relationship with DNMT1a in *B. terestris* and the homologous proteins in *A. mellifera*, *N. vitripennis*, *Z. nevadensis* and *C. floridanus* (Fig. 2A). DNMT2 and DNMT3 in *B. terrestris* presented closer relationships with these of *A. mellifera* (Fig. 2A). Similarly, MBD3 from *B. terrestris* showed a closer relationship with that of *A. mellifera* (Fig. 2B). And phylogenetic analysis suggested TET2 from *B. terrestris* and *A. mellifera* shared closer relationship (Fig. 2C). Above all, DNA methylation-related genes in *B. terrestris* displayed closer relationships with those of other Hymenoptera insects. Hence, the obtained sequences in *B. terrestris* are reliable.

### The comparison of DNA methylation systems between *B. terrestris* and other insects

**Figure 3:**
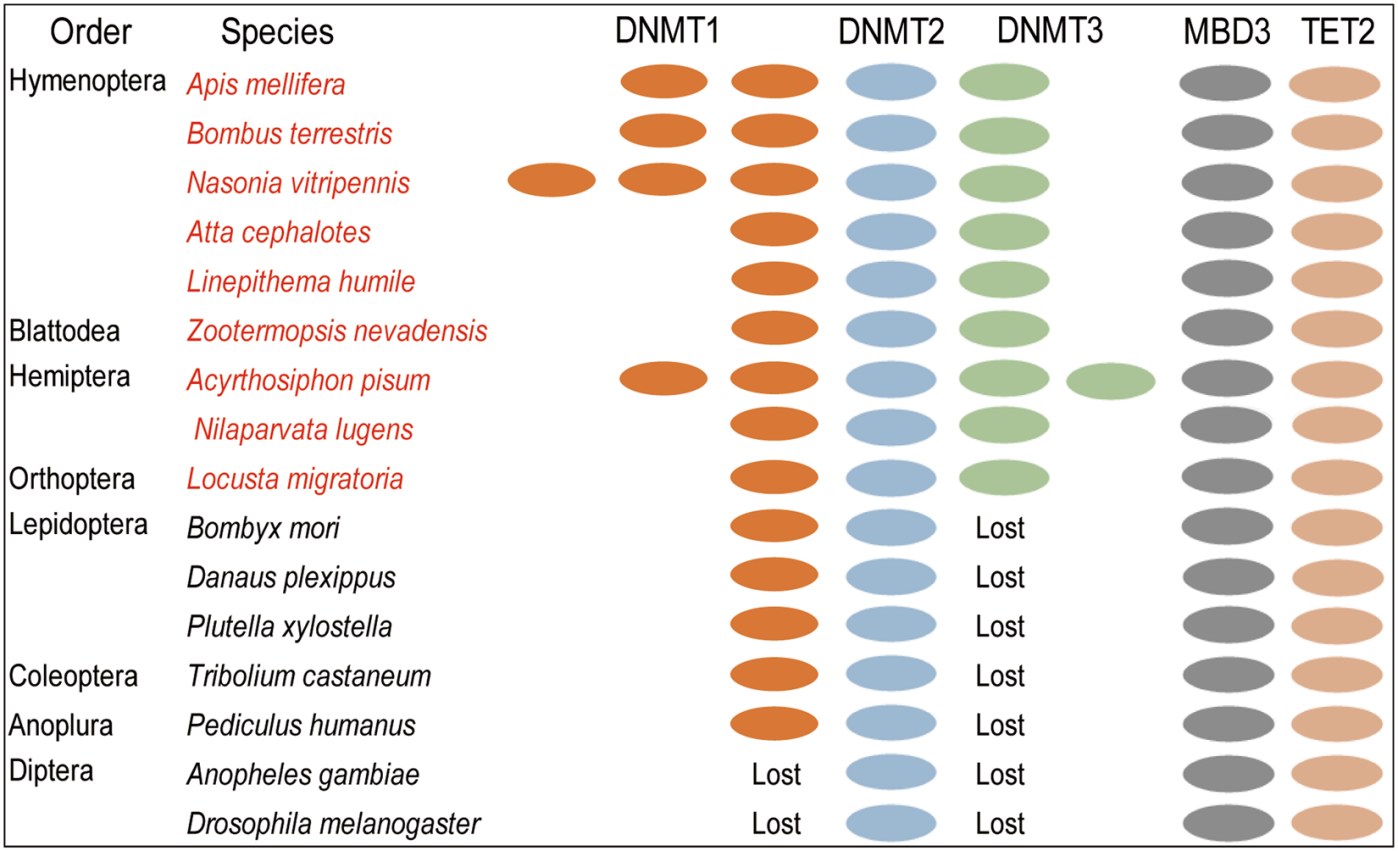
Comparison of DNA methylation systems in insect species. Different insect species display varied DNA methylation systems. The numbers of ellipses of each color indicate the numbers of gene copies found in each species. Lost in figure means that DNMTs are absent in insect genomes. The red marked species in the figure contain a complete functional DNA methylation toolkit.

The DNA methylation process needs a set of essential enzymes including DNA methyltransferases and demethylases^31^. However, DNA methylation-related proteins were selectively lost in insect species. Based on previous researches^25,32^, we revised the patterns of DNA methylation-related proteins in several insects from eight orders, including Hymenoptera, Blattodea, Hemiptera, Orthoptera, Lepidoptera, Coleoptera, Anoplura, and Diptera (Fig. 3). Bumblebees, honeybees, ants and wasps contain full DNA methylation toolkits with different duplications of DNMT1^33–35^. Likewise, *Z. nevadensis* was found to have a full DNA methylation toolkit^36^. Aphides and brown planthoppers also harbor the full functional DNA methylation systems^30,32^. Besides, the locust genome possesses a complete DNA methylation toolkit^37^ (Fig. 3). Nevertheless, DNMT3 was lost in some Lepidoptera, Coleoptera and Anoplura insects as shown in Fig. 3^38–41^. Both DNMT1 and DNMT3 were lost in flies and mosquitos, only DNMT2 was found in their genomes^42^ (Fig. 3). These results suggested that *B. terrestris* has a complete DNA methylation system, providing new insights into the important roles of DNA methylation in social insects.

### Caste-specific expression patterns of DNA methylation-related genes in *B. terrestris*

**Figure 4:**
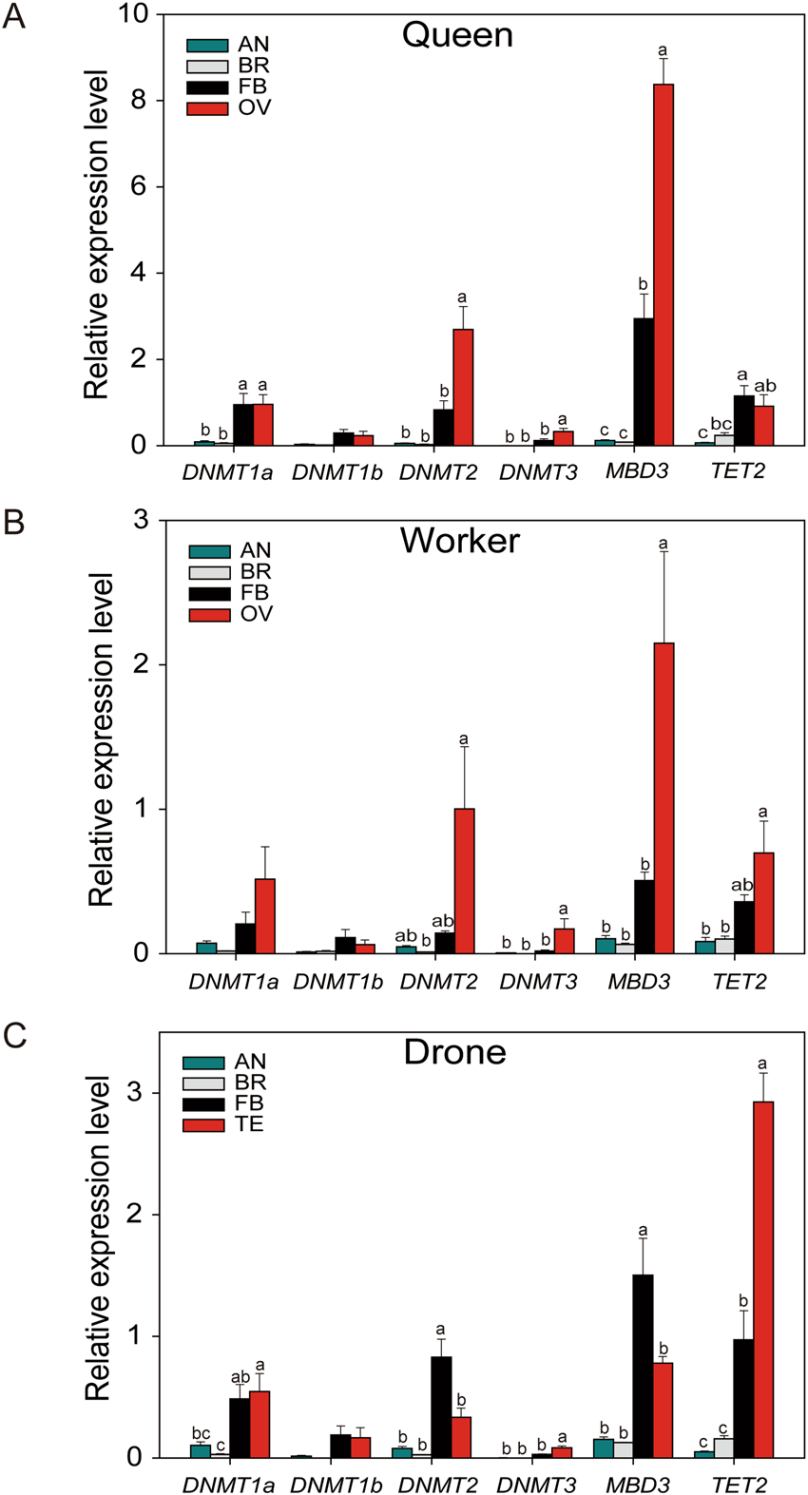
The expression patterns of DNA methylation-related genes of *B. terrestris* between different tissues in queen (**A**), worker (**B**), and drone (**C**) adults respectively. AN: antenna; BR: brain; FB: fat body; OV: ovary; TE: testis. Data in the figure represent mean ± SM of four biological replicates, and the different letters indicate significant difference among different castes by multiple testing in one-way ANOVA model (P < 0.05).

qRT-PCR was performed to investigate the expression patterns of DNA methylation-related genes in different tissues from queen, worker, and drone adults. The results showed that most of these genes were highly expressed in fat bodies, ovaries and testes in bumblebee adults (Fig. 4). In queens, *DNMT1a* showed higher expression levels in fat bodies and ovaries than antennae and brains (ANOVA, F_3,12_ = 11.072, P = 0.0012), whereas there was no significant difference in the expression levels of *DNMT1b* among different tissues (Fig. 4A). The expression levels of *DNMT2*, *DNMT3* and *MBD3* were significantly higher in ovaries than other tissues (ANOVA, *DNMT2*: F_3,12_ = 19.097, P < 0.0001; *DNMT3*: F_3,12_ = 13.393, P < 0.001; *MBD3*: F_3,12_ = 88.284, P < 0.0001) (Fig. 4A). *TET2* showed higher expression levels in fat bodies and ovaries than antennae and brains (ANOVA, F_3,12_ = 7.809, P = 0.004) (Fig. 4A).

In workers, the expression levels of *DNMT1a* and *DNMT1b* showed no remarkable difference among four tissues (Fig. 4B). *DNMT2*, *DNMT3* and *MBD3* exhibited higher expression levels in ovaries than other three tissues (ANOVA, *DNMT2*: F_3,12_ = 3.996, P = 0.035; *DNMT3*: F_3,12_ = 5.289, P = 0.015; *MBD3*: F_3,12_ = 8.405, P = 0.003) (Fig. 4B). *TET2* displayed higher expression levels in fat bodies and ovaries than antennae and brains (ANOVA, F_3,12_ = 6.226, P = 0.009) (Fig. 4B). In drones, *DNMT1a* was highly expressed in testis and fat body tissues (ANOVA, F_3,12_ = 6.838, P = 0.007) (Fig. 4C). While the expression levels of *DNMT1b* showed no obvious distinction in all tissues. Both *DNMT2* and *MBD3* displayed higher expression levels in fat bodies than other three tissues (ANOVA, *DNMT2*: F_3,12_ = 18.972, P < 0.0001; *MBD3*: F_3,12_ = 14.594, P = 0.0003) (Fig. 4C). Relative expression levels of *DNMT3* and *TET2* were significantly higher in testes than other tissues (ANOVA, *DNMT3*: F_3,12_ = 24.640, P < 0.0001; *TET2*: F_3,12_ = 61.465, P < 0.0001) (Fig. 4C).

**Figure 5:**
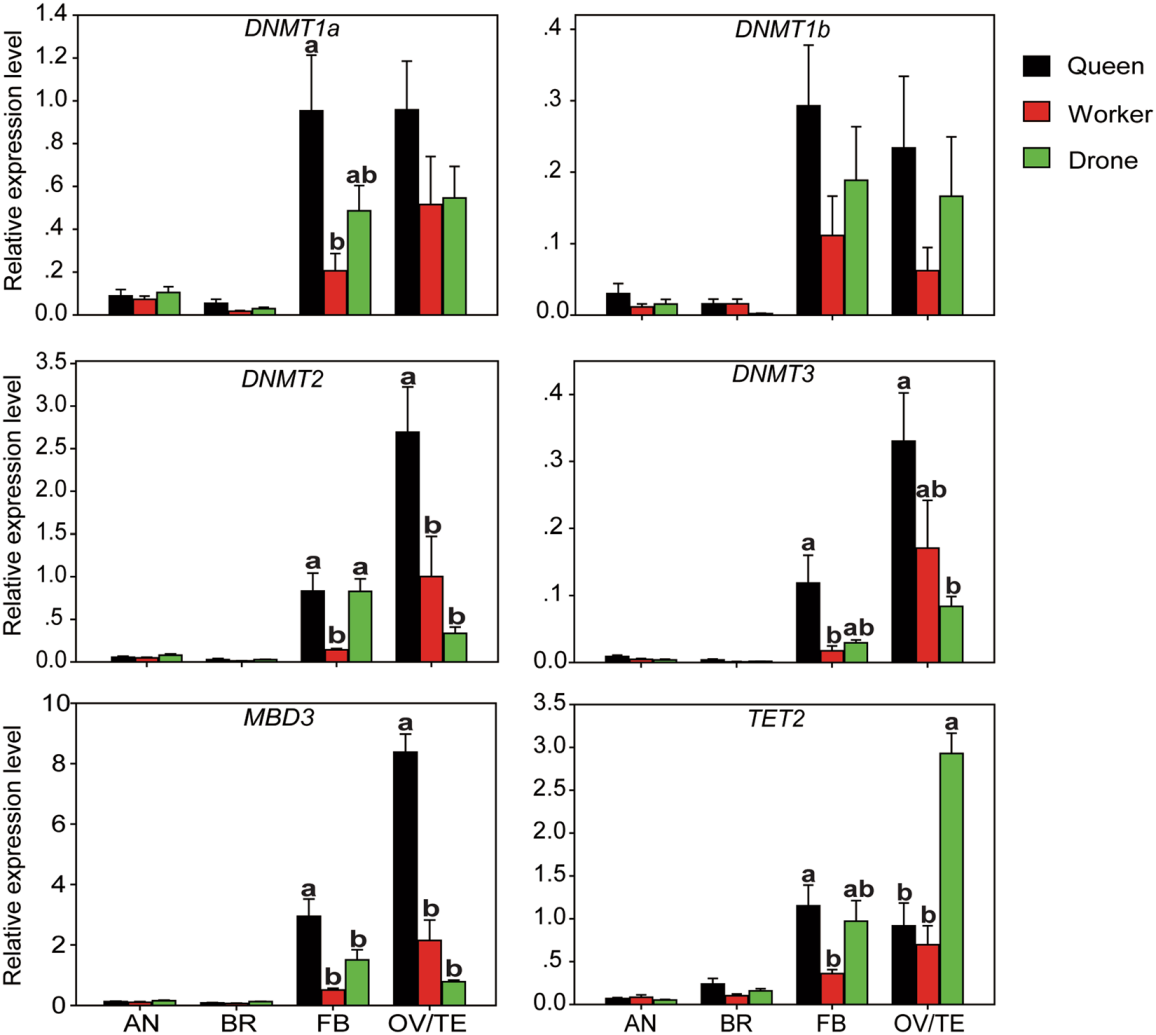
The expression patterns of DNA methylation-related genes of *B. terrestris* in different tissues among queen, worker, and drone adults. AN: antenna; BR: brain; FB: fat body; OV: ovary; TE: testis. Data in the figure represent mean ± SM of four biological replicates, and the different letters indicate significant difference among different castes by multiple testing in one-way ANOVA model (P < 0.05).

In different castes, the expression levels of these genes showed significant differences in fat bodies and gonads but not in antennae and brains (Fig. 5). The expression levels of most genes were significantly higher in fat bodies of queen adults than in those of worker adults (ANOVA, *DNMT1a*: F_2,9_ = 4.839, P = 0.037; *DNMT2*: F_2,9_ = 7.086, P = 0.014; *DNMT3*: F_2,9_ = 5.071, P = 0.034; *MBD3*: F_2,9_ = 10.177, P = 0.005; *TET2*: F_2,9_ = 4.435, P = 0.046). For ovaries, *DNMT2* and *MBD3* were significantly higher expressed in queens than workers (ANOVA, *DNMT2*: F_2,9_ = 8.689, P = 0.008; *MBD3*: F_2,9_ = 59.993, P < 0.0001). There was no significant difference in the expression levels of most genes between females and males in fat bodies (Fig. 5). However, *TET2* exhibited much higher expression levels in male testes than female ovaries, and no obvious difference occurred between ovaries of queens and workers (*TET2*: F_2,9_ = 25.778, P = 0.0002) (Fig. 5). These results indicated that these DNA methylation-related genes displayed apparent caste-dependent expression patterns.

### Expression patterns of DNA methylation-related genes in queenright and queenless workers

**Figure 6:**
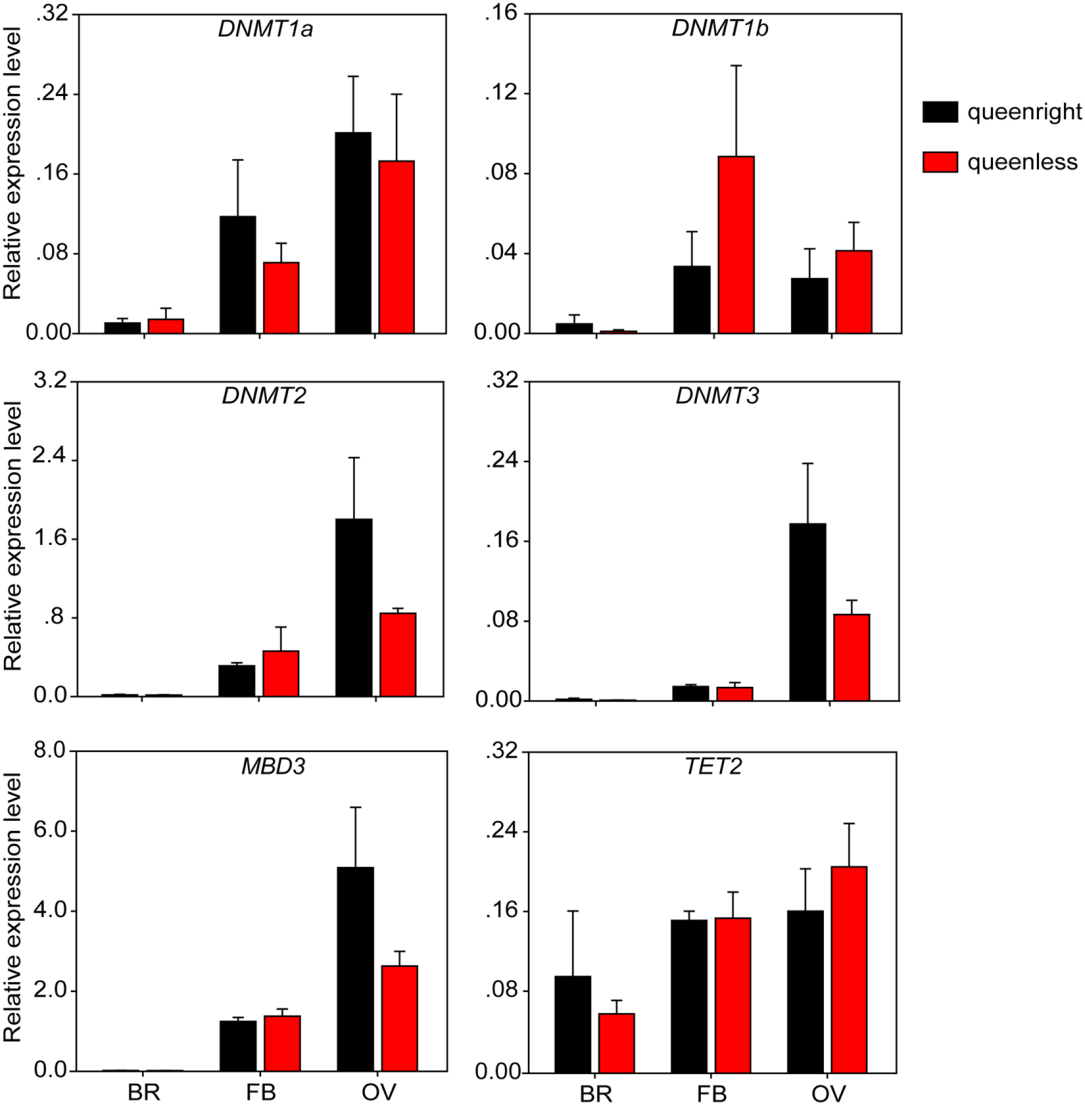
The expression patterns of DNA methylation-related genes in different tissues of workers in queenright and queenless colonies. AN: antenna; BR: brain; FB: fat body; OV: ovary; TE: testis. Data in the figure represent mean ± SM of four biological repetitions. The values between groups were analyzed by independent sample T-test (P < 0.05).

In bumblebees, workers are able to activate their ovaries and lay haploid eggs after the death of the queen or the removal of the queen in a colony^43^. We investigated whether DNA methylation is associated with queen-repressed worker fertility in *B. terrestris*. After measuring the length of terminal oocytes of workers in queenless and queenright colonies, we found that the ovaries in queenless workers were significantly larger than those in queenright workers (Supplementary Fig. S6). The expression patterns of these genes were determined in different tissues, including brains, fat bodies, and ovaries from queenright and queenless workers (Fig. 6). In brains, *TET2* showed a scarcely higher expression level in queenright workers than in queenless workers. In fat bodies, *DNMT1a* displayed a barely higher expression level in queenright workers than in queenless workers. Whereas the expression levels of *DNMT1b* and *DNMT2* were slightly higher in fat bodies of queenless workers than in those of queenright workers (Fig. 6). For ovaries, *DNMT1a*, *DNMT2*, *DNMT3*, and *MBD3* showed marginally decreased expression levels in queenless workers compared with those in queenright workers. In contrary, the expression levels of *DNMT1b* and *TET2* were slightly higher in ovaries of queenless workers than in those of queenright workers. However, no significant difference was observed in the expression levels of these genes between queenright and queenless workers (Fig. 6).

## Discussion

In this study, we identified six genes involved in DNA methylation in bumblebees. These genes exhibited caste-specific expression patterns in *B. terrestris*. No remarkable difference in the expression levels of these genes was observed between queenright workers and queenless workers. This study provides molecular insights into the DNA methylation-related gene system of *B. terrestris*.

Our results revealed that there is a complete DNA methylation system in *B. terrestris*, similar with other social insects, aphids, and locusts (Fig. 3). Actually, DNA methylation systems display diverse patterns among insects. For example, *D*. *melanogaster* lacks two key DNMTs (DNMT1 and DNMT3), and a very low level of cytosine methylation exists at the early embryonic stage^44^. Homologous genes of *DNMT1* and *DNMT2* were discovered in *B. mori* genome, but no homologous gene of *DNMT3* was detected^38^. Similarly, *T. castaneum* lacks *DNMT3*, and it contains a significantly lower DNA methylation level^45^. However, the complete functional DNA methylation toolkit was observed in most of social insects. Social insects usually exhibit a striking environment-driven plasticities including morphological, reproductive and behavioral diversities from the same genome^46^. Epigenetic mechanisms are widely considered additional aspects of genome regulation for increasing flexibility of organisms^47^. Therefore, the conservation of functional DNA methylation systems in these social insects suggests that DNA methylation may be associated with social evolution.

DNA methylation-related genes displayed obvious caste-dependent expression patterns in *B. terrestris*. A previous large-scale RNA-seq analysis from 11 different combinations of caste and developmental stages in *B. terrestris*^48^, also implied that possible differential expression patterns of DNA cytosine methyltransferase genes in different castes. And *DNMT3* shows different expression patterns between queens and workers in the honeybee *Apis cerana cerana*^49^. Besides, a number of documents have reported that DNA methylation is widespread and associated with caste differences in many social insects, including *Z. nevadensis*^17^, *C. floridanus*^50^, *Harpegnathos saltator*^50^ and *A. mellifera*^15^. We also found that most DNA methylation-related genes were higher expressed in fat bodies and ovaries of queen adults than in those of workers and drones in *B. terrestris*, implying that DNA methylation processes bears important functions in caste determination.

In mammals, relative mRNA expressions of *DNMTs* have been reported to be associated with the global dynamic of DNA methylation status^51^. DNA methylation, as an important epigenetic factor in regulations of gene expression and transcriptional status^52^, may plays vital roles in physiological processes relies on the participation of DNMTs. Higher *DNMTs* expression levels existed in fat bodies and ovaries of adult queens imply that more active methylation processes may occur in these two tissues of queens than in those of workers, which is consistent with a large demand of energy substances to be accumulated in fat bodies and ovaries for ovary development and egg laying in the queen^53^. These results suggest that DNA methylation may be closely related to the development of ovary and reproduction of the queen in the bumblebee.

In the present study, most genes in drones displayed intermediate expression levels compared to the two female castes. Although females and males differ in their ploidies, they share the same genome. The differential expression patterns of these genes implied distinct DNA methylation status in these three castes in the bumblebee. These wide difference of DNA methylation may be associated with unique roles of specific castes played in the bumblebee colony. However, more research is needed to understand the mechanisms underlying the result.

Our results showed that *TET* displayed the highest expression level in drone testes. In mammals, three members of TET proteins have been reported to be involved in the demethylation process^28^. Until now, only one member of TET enzymes has been identified in insect genomes^31^. In the honeybee *A. mellifera*, TET dioxygenase has been reported to possess catalytic activity to convert 5-methylcytosine to 5-hydroxymethylcytosine^54^, suggesting similar functions of insect TET enzymes with that of mammals. It was reported that DNA methylation dynamics are crucial for the differentiation of spermatogenic cells in mammals^55^. During spermatogenesis, TET proteins are involved in the regulation of maintenance of pluripotency and proliferation of male germline stem cells^56^. The highest expression level of *TET2* in drone testes of bumblebees implied potentially active demethylation in male gonads. Therefore, TET-mediated demethylation may be necessary for spermatogenesis in bumblebees, however, the detailed functions of TET should be further determined.

We found that no difference was observed in the expression levels of DNA methylation-related genes between queenless and queenright workers in *B. terrestris*. Bumblebee workers are well acknowledged to show remarkable reproductive plasticity, depending on social context. After the competition point in queenright colony, workers were not inhibited by the queen and then became fertile^57^. Influence of queen on worker reproduction constantly has been an interesting question, which presents great significance to evolution of social insects. Many studies suggested that chemical pheromones and direct contact with the queen play important roles in regulating reproductive dominance in bumblebees^58,59^. Several molecular mechanisms, such as epigenetic regulations, also were proposed to determine plasticity of worker reproduction^60,61^. However, differential expression levels of genes related to DNA methylation were not observed between queenright workers and queenless workers, implying that DNA methylation may not be directly involved in queen-inhibited worker reproductions. The result is in agreement with a recent study which reported that levels of DNA methylation exhibited no difference between queenright nonreproductive workers and queenless reproductive workers^62^. Therefore, differential expressed genes involved in DNA methylation may mainly be involved in caste differentiation, but not in worker reproductive plasticity depending on the social context.

DNA methylation serves as a complex dynamic system involved in regulating many important biological processes in insects. In this study, we focused on the expression patterns of genes related to DNA methylation in bumblebee adults. Functional studies on DNA methylation system in different developmental stages should provide significant discoveries for exploring mechanisms of caste differentiation in bumblebees.

## Methods

### Insects

*B. terrestris* was bought from the breeding base of pollinating bees, Information Institute of Beijing Academy of Agriculture and Forestry Sciences, and reared with pollen and 50% sucrose water at 28 °C and relative humidity of 50–60% under red light in well-ventilated plastic boxes.

### Sequence analysis

Nucleotide sequences of genes were obtained from the National Center for Biotechnology Information with blast tool. Putative ORFs and amino acid sequences of DNA methylation-associated genes in *B. terrestris* were predicted using DNAMAN software. Protein properties were analyzed with EXPASy server, and protein domains were predicted with SMART software^63^. Sequences used in phylogenetic analysis were downloaded from the National Center for Biotechnology Information gene database (http://www.ncbi.nlm.nih.gov). DNA methylation-associated genes of *B. terrestris* and other selected insects were subjected to multiple sequence alignments using Clustal W program^64^ and edited with GeneDoc software^65^ (Supplementary Figs S1–S5). The phylogenetic trees were constructed using a Maximum Likelihood method with a Poisson model and 1000 Bootstrap replications of MEGA5.0 software^66^.

### Sample preparation

Different tissues (antenna, brain, fat body, ovary, and testis) of *B. terrestris* were dissected from one-day-old adults that from queens, workers and drones. Wherein adult workers, virgin queens and drones were obtained from four colonies separately. Four biological replicates were prepared for each experiment, and each biological replicate included eight individuals. Collected samples were immediately stored at −80 °C for further total RNA extraction.

Five newly emerged workers were placed in a new nest containing the mother queen from the native colony, as a queenright treatment. In queenless treatment, five newly emerged workers were selected and reared in a new colony without the queen. There were four biological replicates in each treatment. Five days later, different tissues (brain, fat body, and ovary) were dissected from workers in queenright and queenless colonies. Harvested samples were kept in −80 °C at once to extract total RNA.

### RNA extraction and cDNA synthesis

Total RNAs were isolated using a TRIzol kit (Life Technologies) following the manufacturer’s instructions. Quantity and quality of total RNA were determined by ultraviolet spectrophotometry. Before first-stand cDNA synthesis, DNase was used to treat total RNA to prevent genomic DNA contamination. First-strand cDNA was synthesized from 2 μg total RNA using the M-MLV Reverse Transcriptase and oligo (dT)-primer (Promega) according to the manufacturer’s instructions. Synthesized first-stand cDNA was stored at −80 °C immediately for subsequent use.

### Quantitative RT-PCR (qRT-PCR)

Primers used for qRT-PCR were designed by Primer 5.0 software, and are listed in Supplementary Table S1. The gene *Elongation Factor 1a* (*EF1a*) in *B. terrestris* was used as a reference gene for normalization in accordance with the previous study^67^. The cDNA was subjected to qRT-PCR by using the SYPR Green RealMasterMix (Roche) according to the manufacturer’s instructions on a LightCycler 480 instrument (Roche). Melting curve analysis was used to confirm unique amplification. Data were analyzed by 2^−ΔΔCT^ method to quantify relative mRNA expression levels^68^.

### Statistical analysis

Data in figures are expressed as means ± standard error (M ± SM), and qRT-PCR results were analyzed using multiple comparison in one-way ANOVA and independent sample T-test by SPSS17.0 software.

## Electronic supplementary material

Supplementary Information
