# Supplementary material for: Identification and caste-dependent expression patterns of DNA methylation associated genes in Bombus terrestris
Source: Sci Rep. 2018 Feb 5;8:2332. doi: 10.1038/s41598-018-20831-1 (PMC5799256; doi:10.1038/s41598-018-20831-1)
Supplement: Supplementary file 1 — Supplementary Information [file 41598_2018_20831_MOESM1_ESM.pdf]

Supplementary Information

Identification and caste-dependent expression patterns of DNA methylation associated genes in *Bombus terrestris*

Beibei Li[1], Li Hou[2], Dan Zhu[2], Xilian Xu[3], Shiheng An[1]* & Xianhui Wang[2]*

[1]College of Plant Protection, Henan Agricultural University, Zhengzhou 450002, China. [2]Institute of Zoology, Chinese Academy of Sciences, Beijing 100101, China. [3]Institute of Plant and Environment Protection, Beijing Academy of Agriculture and Forestry Sciences, Beijing 100097, China. *Corresponding authors. Email address: anshiheng@aliyun.com (S.-H, An) and wangxh@ioz.ac.cn (X.-H, Wang)

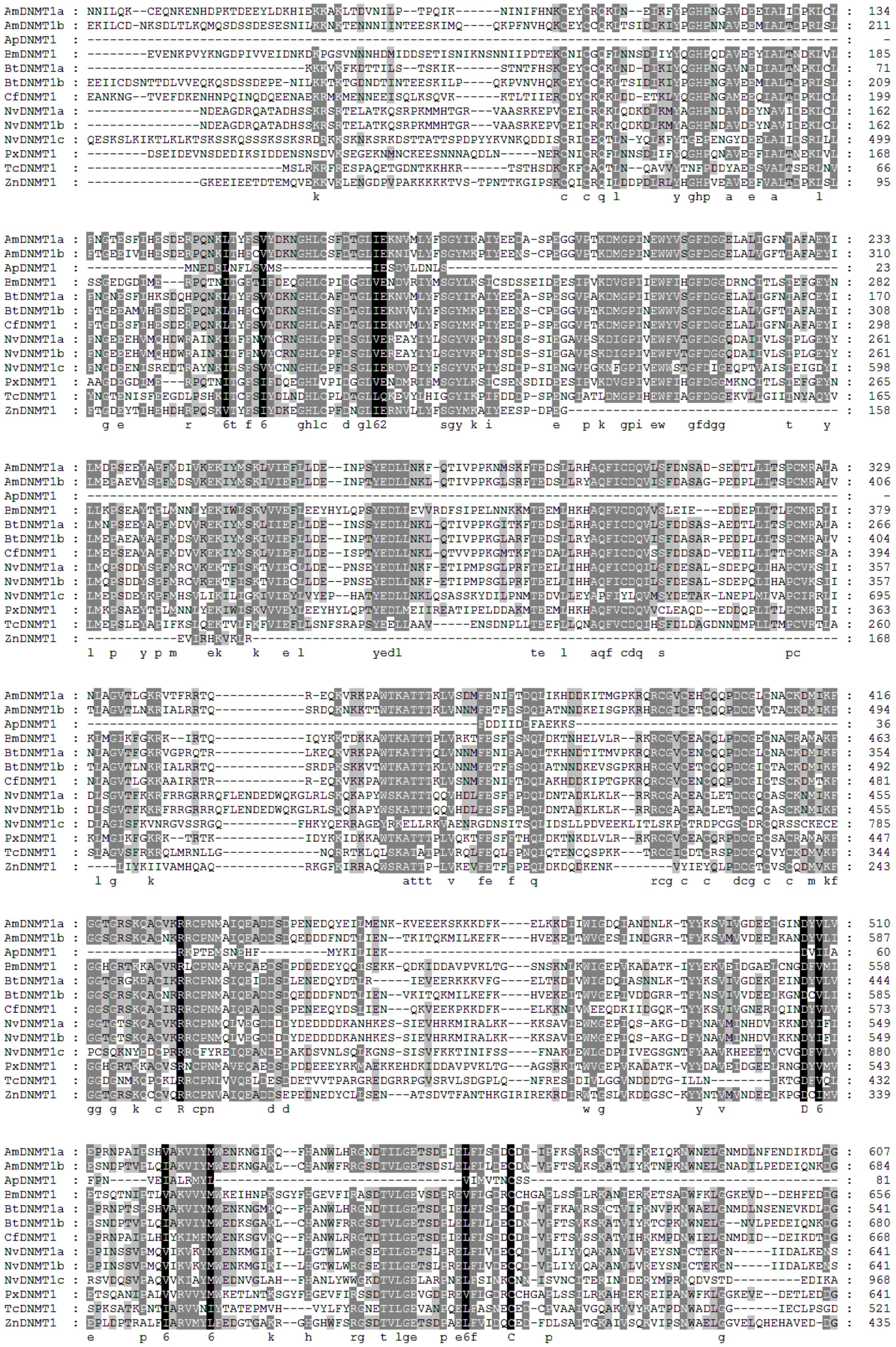

```
AmDNMT1a : KTFFFQKRYIPETARFEDPVPDVKYLHKKNSYRFCPACTRLNMLEQFNTPKVYERI-----EEINSKEVIYGLVKYKGEDYRVGTTVFLQPDVFNFKYKT :  702
AmDNMT1b : KTFFYQKKYIPETARFEDCSLDPECPRKEISHRFCPACIRFTTLQCYYTPKVFDRE-----EEKNSKEVTYNIVKYKNEEFKVGSAVFLMPGAIKYKYTS :  779
ApDNMT1  : --------------------------------------------------ERTFSRL-----------KIIKNRLRTTMNEDRLN---FLS---------- :  108
BmDNMT1  : RTYFYSKYYDRFTSRFEDLPPDPACPNALRKHRFCPSCERKTKRDARNIPKVFEKLIVKSEIVSEQNRSEWSYVKWQDFDYKKGCGVFLKPGTFKLKNSM :  756
BtDNMT1a : KTFFYQKRYTPATARFEDPLPDLICPFKEKSHRFCPACAHLREVEQFNTPKVYERI-----KEKSTKEVIYGLVKYKGEDYRVGTTVFLQPKAFNFKYKI :  636
BtDNMT1b : KTFFYQKKYTPETARFEDCTSDPECPRKEISHRFCPACVRFTTLQCYYTPKVFNRE-----EEKSNKEVTYSMVKYKGEEFRVGSTVFLMPAAIKYKFTS :  775
CfDNMT1  : KTFFYQMRYTPETARFENSLPDPECPRKENSHRFCPACARLRALEQYNTPKVFERI-----EEKSSKEVIYGVVRYKDEEYRVGSAVFLYPSAFKFKYKQ :  763
NvDNMT1a : MILFYQKRYDHVAARFEDLLPELN-PPKGAEHCFCSTCARDILSKMNRTPQLLELLD-----DTDDKRIKYGIIRYLNEEFRVGSAVYLKPKTFTFEFPN :  735
NvDNMT1b : MILFYQKRYDHVAARFEDLLPELN-PPKGAEHCFCSTCARDILSKMNRTPQLLELLD-----DTDDKRIKYGIIRYLNEEFRVGSAVYLKPKTFTFEFPN :  735
NvDNMT1c : NNIFCEKSYDPSDGSFHDLTKPEE-VSVLPTHRSCINCEGKHRRSERVEPRTVDELG------KKEEGTLYRAVIYKDEEYIVGSCVYLKPKSLYFQFPM : 1061
PxDNMT1  : KTYFYKKYYERFTARFEDLPEDPACPNPLRQHRFCPSCERKTRKDAKNIPKVIDKLAEKSEFVTEANRTEWSLVRWQDHDYKRGGGVFLKPGTFNLKSSL :  741
TcDNMT1  : DRFFYAKQYDSETATFTDYVKVRN------ELDSCGHCRLNEERKKIETPTFKDNK-----------------VQWRNEFYEAGNSVFLDPSVYQFPIPV :  598
ZnDNMT1  : KTFFTQKQYDWIHARFEDLAADPPC-LEELRYRFCASCERSCLQQQYRRPKIYEKLG----VTNENHEVLFGVVRLRGEEYRVGTSVFLQPGSFKFKTSG :  530
              f  k y    arfe                fc  c           p                       6    e    g  v5L p

AmDNMT1a : TYQDVLK--LKKENVDEDMYPEFYRKSSDHIKGSNFDTPDPFCIGYINKIFANTNDMLVSPSDINIMVNKLYRPENTHKSSTLMEQADLNMVYWSNEVCN :  800
AmDNMT1b : TYHISQK--VKKGKVDEDMYPEYYRKSSDHVKGSNYDTPEPFHIGYINSIYATTNNKLVASSDIWIKINKMYRPENTHKGLTLMQQVDLNMVYWSDEVCD :  877
ApDNMT1  : -------------------------------------------------------------------------------------------VMSIESDVLDN :  119
BmDNMT1  : TKANTVAK-PRFEKVDETIYPEYYRKNDSNTRGSNIDTGEPFCVGYIAAVTAASEGPLVVPQDIYLKVNVLLRPENTSS--KFPQHEDTNVLYWTTEIRE :  853
BtDNMT1a : IYPDAQK--VKKENVDEDMYPEFYRRSSDHTKGSNVDTPDPFCIGYINEIYASTNDMLVAPSDINIKVNKLYRPENTHKYLTLMEQADLNMVYWSDEVCN :  734
BtDNMT1b : TYHISPK--VKKGKVDEDMYPEYYRKSSDHVKGSNCDTPEPFHIGYINSIYATTNNKLVASSDIWIKINKLYRPENTHKGLTLMQQVDLNMVYWSEEVCD :  873
CfDNMT1  : MYQEAEK--PKKEVVDEDMYPEFYRKVSEKAKFSNVDTPEPFHIGYINAIYASTTDLIVSSSDIYIKVNKMYRPENTHRDVTLMEQADLNMVYWSEEICS :  861
NvDNMT1a : MNQDTSRG-QKREIVDEEKYPEAYRKFNDRVKGSNVDTPEPFDIGYITSIYSTSKSKLLAGVNAYVTVKKMYRPENTHRGESLKKKSDMNMLYWSDEECD :  834
NvDNMT1b : MNQDTSRG-QKREIVDEEKYPEAYRKFNDRVKGSNVDTPEPFDIGYITSIYSTSKSKLLAGVNAYVTVKKMYRPENTHRGESLKKKSDMNMLYWSDEECD :  834
NvDNMT1c : LLDHAKSSGKISKKIDEEKYPELYRKAEDAVIDSKFDMPAPFDIGYITEIFTT--------DKLYIKVKKLYRPENTHRNEVLIKQNDMNMLYWSNEVCT : 1153
PxDNMT1  : TYS-DTSK-PKLENVDESVYPEYYRKSDNNLRGSNVDTGEPFCVGLIVAAAARGAGPLVVPQDIYLRVRVMCRPEHTAS--RFPQHEDLNLVYWSDDIRE :  837
TcDNMT1  : ECTFDDAK-----EMNESKYPEYYRKREAN--ASCKKMPQPFCIGLIETLCDG-------PAGVLMAIRVFFRPENTKGGSMLSYQSDINLLFWSTKFIT :  684
ZnDNMT1  : TAAVTKKDKSKDEKVDEDVYPEFYRKSSDHVRGSNMETPEPFCVGHIKTIFAKTNDNLVPSRDIWIRVNKFYRPENTHRGPCLGQQMDLNMLYWSEEECV :  630
                           e  ype yr        sn dtp pf  g i                         rpent     l    d n6  w3 e

AmDNMT1a : IKFIKVVGKCYLTYSEN--LNQSIEEWIAAGPYRFYFNEAYNVQEKTFDEPPYHAISIGKC--GKSKGNLKFK---GKKTEESEKRAIVNTPIEYKKISE :  893
AmDNMT1b : IKFSDVAGKCYLVYSEN--LDQSVEEWTTAGPYRFYFSQAYNASEKTFVDPPYYAMNIGKLGSGKGKGKAKCK---SKKLETSDKK-IIDKPINYCKVPK :  971
ApDNMT1  : LSFDDIID-----------------------------------DFAE----------------KKSRK-------------------------------- :  136
BmDNMT1  : IPFSTVVGHCHLIYEQNVPQNISLQEWLGNDPCRFYFRMAYCKSTGEFTDLPQNAISVGRTDRTKDKGKGKGK---------STKTIETVPAKVVEEKIR :  944
BtDNMT1a : IKFIEVVGKCYLAYSEN--LNQTVDEWTAEGPNRFYFNEAYNAQEKTFNEPPYHAISIGKS--GKSKGNLKFK---GKKTEESKKRAFVNRPIEYNKILK :  827
BtDNMT1b : VKFSDVAGKCYLAYSEN--LDQSVEEWTAAGPNRFYFSQAYNALEKTFVDPPYYAMNIGKSVIGKGKGKAKCK---SKKLENSDKK-IVDKPIDYCKVIR :  967
CfDNMT1  : VKFSEVAGKCYLAYSEN--LSESVEEWSLGGPNRFYFTEAYNAKENSFDEPPYNVINIGKS--GKGKGKGKSK---AKKVGETENKPVIDKPIDHPVVIR :  954
NvDNMT1a : VRLNCIVGKCYIAYSEN--LNQSIEEWSASGPHRFYFSEAYDLNNEEYTEPPAHACSVTKSFKKDVKAKSKSK---------KVETIIEDIPVSLPQISH :  923
NvDNMT1b : VRLNCIVGKCYIAYSEN--LNQSIEEWSASGPHRFYFSEAYDLNNEEYTEPPAHACSVTKSFKKDVKAKSKSK---------KVETIIEDIPVSLPQISH :  923
NvDNMT1c : VSFNYVVRKCYVAHIKD--FSKSVEEWSNLGPDRFYFMQMYHDKKFTDIERNMYGCEDVPLDPPIDYPDVDAMT------------------------- : 1225
PxDNMT1  : VPFSAVVG-----------------------------KTGKFTEVPPEAESIGRIDKGKEKGKGKGK---------SSKASATT-ETSANASVR :  892
TcDNMT1  : VPFRKVMGKCYVVYCSD---EQKAREWSEGGPYRYYFRQQYDPDCGELVPPTYSCTRLALR-----EGAGQDE---------------------RPFGR :  754
ZnDNMT1  : LNFSEVVGKCHVVFSEN--VDIPMAEWSSHGPYRFYFCQAYNSIKQTFEEPPFHATNIGMYGKGKGKGKGKGKKTWSETEEEKKKKKNGMKIEDWPQNHT :  728
           6 f  6 gkc               ew   gp r yf   y          p              k   k k

AmDNMT1a : KLKTLDVFAGCGGLSEGLHQAGVAENL--WAIEKEESAAYAYRLNNPNATVFIEDCNVLLKKVMNGETTNEIGQKLPQKGQVELLCGGPPCQGFSGMNRF :  991
AmDNMT1b : KLKTLDVFAGCGGLSEGLRQAGIVDNQ--WAIEKDEPAACAYRLNNPNTTVFCEDCNVLLRKVMNGDLCDNNGQRLPQKGEVELLCGGPPCQGFSGMNRF : 1069
ApDNMT1  : -------FAGCGGLSRGLEDSGLVXSN--WAIECDDKAAGAFKLNNPEATVFVEDCNHLLKLAMAGEKSNSKNQNIPQKGEVDFICGGPPCQGFSGMNRF :  227
BmDNMT1  : PLRTLDVFAGCGGLSEGLHQAGVAECK--WAIENVEAASHAYSLNNKSCIVFNEDCNALLKTVMSGAKHSANGLRLPMQGEVELLCGGPPCQGFSGMNRF : 1042
BtDNMT1a : KLRTLDVFAGCGGLSEGLYQAGITENL--WAIEKEEAAANAYRLNNPNATVFTDDCNILLKMVMDGEKTNKVGQKLPQKGQVELLCGGPPCQGFSGMNRF :  925
BtDNMT1b : KLKTLDVFAGCGGLSEGLRQAGIVDNQ--WAIERDEPAACAYRLNNPNTTVFCEDCNVLLRKVMKGDLCDNNGQRLPQKGEVELLCGGPPCQGFSGMNRF : 1065
CfDNMT1  : KLRTLDLFAGCG---------------------------------------------------------------------------------------- :  966
NvDNMT1a : KLRTLDVFAGCGGLSEGLKQAGVAESL--WAIENDTAAAHAYRLNNPKASVFTTDCNSFLEKVINGETSLG-GQSLPKKGEVDLLCGGPPCQGFSGMNRF : 1020
NvDNMT1b : KLRTLDVFAGCGGLSEGLKQAGVAESL--WAIENDTAAAHAYRLNNPKASVFTTDCNSFLEKVINGETSLG-GQSLPKKGEVDLLCGGPPCQGFSGMNRF : 1020
NvDNMT1c : ---SLEIFAGCGGLSLGLRESGVIKKLGSWAIESDVDAANTFQLNNPDITVLVGHPDIVLKNAMKGEFEDSDGKLLPRKDDVEFLCATLPCENYKTMT-- : 1320
PxDNMT1  : PLRTLDVFAGCGGLSLGLHTAGAASPR--WAVEQVEPAAHAYELNNRACTVF--------------------------PGEVELLCGGPPCQGFSGMNRF :  964
TcDNMT1  : PLKCLDVFAGCGGLSQGFHAAGVANTK--WAIENDKPALDTFRHNNRTCHVFRDDCNVLLRNVMSGK------GGLPPKSEVEMIVGGPPCQGFSGMNRF :  846
ZnDNMT1  : KLQSLDVFAGCGGLSEGLCQSGVAESC--WAIEKEESAAHAFRLNNPECTVFTEDCNQLLKLALSGEKTNDKGQCLPCKGEVELLCGGPPCQGFSGMNRF :  826
            l  ld FAGCGgls gl   g       wa e    aa a  lnn    vf  dc   l     g          p kg v   cggppc g sgmnrf

AmDNMT1a : NSRQYSLFKNSLVVSYLSYCDYYRPNFFIMENVRNFVSFKRSMVLKLTLRCLIRMGYQCTFGILQAGNYGIPQTRRRMIILAAAPGQMLPKYPEPSHVFS : 1091
AmDNMT1b : NSRQYSLFKNSLVVSCLSYCDYYRPKFFIMENVRNFVSFKRSMVLKLTLRCLVRMGYQCTFGILQAGNYGIPQTRRRLIILAAAPGEVLPKYPEPTHVFS : 1169
ApDNMT1  : NSGQYSLFKNSLIVSFLSYIDFYRPKYFVMENVRNFVSFKRSMVLKLTLRCITRMGYQCTFGILQAGNFGVPQTRRRLIIMAAAPGEKLPLYPEPIHVFN :  327
BmDNMT1  : NSREYSNFKNSLVASYLSFCDYYRPKYFILENVRNFVAFKKGMVLKLTLRALLDMGYQCTFGILQAGNYGVPQTRRRLIILAAAPGYNLPFYPEPTHVFS : 1142
BtDNMT1a : NSRQYSLFKNSLVVSYLSYCDYYRPNFFIMENVRNFVTFERSMVLKLTLRCLVRMGYQCTFGILQAGNYGIPQTRRRIIILAAAPGQILPQYPEPTHVFS : 1025
BtDNMT1b : NSRQYSLFKNSLVVSCLSYCDYYRPKFFIMENVRNFVSFKRSMVLKLTLRCLLRMGYQCTFGILQAGNYGIPQTRRRLIILAAAPGEVLPKYPEPTHVFS : 1165
CfDNMT1  : ------------------------------AQLFLHFD------------------------------------------------------------- :  974
NvDNMT1a : NSRAYSSFKNSLIVSFISFCDYYKPRFFLMENVRNFVSFKKSAVLKLTLSCLSRMGYQCTFGILQAGSYGIPQTRRRMILIAAAPGEILPRFPNPLHVFS : 1120
NvDNMT1b : NSRAYSSFKNSLIVSFISFCDYYKPRFFLMENVRNFVSFKKSAVLKLTLSCLSRMGYQCTFGILQAGSYGIPQTRRRMILIAAAPGEILPRFPNPLHVFS : 1120
NvDNMT1c : ---DITVFKHSYIATFLSYCDYYKPSLFILEADEKLIKRS--FVLKLTLACLVSMGYQVTFDLMQVGCYGAPQNRRRSVLLGAAPDSVLPKFPERLHVFP : 1415
PxDNMT1  : NQREYSNFKNSLVASYLSYCDFYRPKYFILENVRNFVAFKKGMVLKLTLRTLLDMGYQCTFGILQAGNYGVPQTRRRLIILASAPGYKLPLYPEPTHVFS : 1064
TcDNMT1  : NEGEYSLFKNSLVVSLLSLCDYYRPLIFVLENVRNFMLYKGGLILKLTLQCLVAIGYQVRISIVEAGEFGVPQARRRFILIASAPGYQLPRIPEPQHVFL :  946
ZnDNMT1  : NSRQYSLFKNSLIVSYLSYCDFYRPRFFILENVRNFVSFKRSMVLKLTLRCLLRMGYQCTFGVLQAGNYGVPQTRRRAIILAAAPGEILPNYPEPTHVFN :  926
           n   y  fknsl     s cd y p  f  envrnf6  k    lkltl c    gyqctfg   ag  g pqtrrr    aaapg  lp  pep hvf

AmDNMT1a : KRACQLSVLVDNKKYSSNCNWIDSAPFRTISVKDAMFDLPEIKNGWNKEEMSYTN-EPTTHFQRKMRG---KQYQPLLRDHICKEMTPLVEARMAHIPIA : 1187
AmDNMT1b : KRACQLSVIVDNKKYSSNCDWTESAPYRTISVRDAMSDLPNIRNGWNNEEIAYSD-EPISHFQRKVRP---KEPDAILRDHICKDMAPLVEARIAHIPTA : 1265
ApDNMT1  : RRSSSLTVQIGTKKFKTNCKYDESAPMRTVTVYDAWSDLPEIPNGANDEDIIYKS-KPITHLQKLLRYPDNRYAESILSDHICKDMSPLVQARMALIPIC :  426
BmDNMT1  : RRACTLTTTIDGKRFSTNIHWDESAPKRTCTIQDAMSDLPQICNGANKIEIEYGS-MPESHFQRLVRS---NDENSKLRDHICKNMAPLIQARISRIPTT : 1238
BtDNMT1a : KRTCRLSVLVDNKKYSTNCSWTESAPFRTINVRDALFDLPEIKNGWNKEEMSYTN-DAISHFQRRIRG---KQYQPLLRDHICKEMTPLVETRIAHIPVA : 1121
BtDNMT1b : KRACQLSVIVDNKKYSSNCDWIESAPYRTISVRDAMSDLPNIRNGWNTEEMAYSD-EPISHFQRKVRP---KEPDAILRDHICKDMAPLVEARIAHIPTA : 1261
CfDNMT1  : ------------LIYLSNCVWTDSAPFRTITVKDAMCDLPDIKNGWNKEEMSYGS-EPLTHFQRKMRG---KQYQPILRDHICKEMAPLVEARMSHIPTA : 1058
NvDNMT1a : KSTCQLTVDVDNVKYFTFTDVHESAPYRAITVYDALSDLPKIKSGSNEVVMRYEN-DPLTHFQRKMRSG--MDDELALYDHICKDLGPLVEARMGLIPTK : 1217
NvDNMT1b : KSTCQLTVDVDNVKYFTFTDVHESAPYRAITVYDALSDLPKIKSGSNEVVMRYEN-DPLTHFQRKMRSG--MDDELALYDHICKDLGPLVEARMGLIPTK : 1217
NvDNMT1c : KPLCELGIVIDSKKYTQDTFWTESAPYRSVTVQDALIDLPTTKVAIT-EEMNYDA-KSTSLFSKIMRLN---MLEPTVFNHVCKDLGPLVECRISQIPLA : 1510
PxDNMT1  : RRACSLTAAIDGKRFVTNIQWNE-APRRTCTIRDAMSDLPPISNGANQLEREYGS-MPETHFQRLVRAG--ATDETKLRDHICKNMAPLIQARIARVPTT : 1160
TcDNMT1  : QRGSRLDIYVDGVKYTNGNFWTQSAPYRMLHVRDAIADLPAIEHDDNRPQMPYDDDEGTSHFQRKMRG-----NQEELLDHICKPIAPIVQTRIKLIPPK : 1041
ZnDNMT1  : PKVCQLSVMVDDMKFLSNCNWVTSAPFRTITVRDAMSDLPEIQNGQKRDEIPYGG-EPMSHFQRLIRG---NQYQPILRDHICKDMAPIIEARIAHIPTA : 1022
              c l    d   5        sAP R   6 DA  DLP i  g n     Y    p 3hfq4 6R          6 1H6CK 6 P662aR6  6P

AmDNMT1a : SGSDWRDLPNIAVRLSDGTYCKQLEYTHDDKKAGRSSVGAYRGVCCCCTGKSCDPTDRQFNTLIPWCLPHTGNRHNHWAGLYGRLEWNGYFSTTITNPEP : 1287
AmDNMT1b : SGSDWRDLPNITVRLSDGTYSKKLEYMYHDKKAGKSSTGALRGVCSCCMGKMCDPIARQYNTLIPWCLPHTGKRHNHWAGLYGRLEWDGFFGTTITNPEP : 1365
ApDNMT1  : EGSDWRDLPNITVQLPEGLKTSKLLYTHHDIKNGYGPNGALRGVCTCASGDKCDPQDRQNNTIIPWCLPHTGNRHNNWAGLYGRLAWSGFCSTTITNPEP :  526
BmDNMT1  : PGSDWRDLPNISVTLSDGTKCKVLQYRYDDKRNGRSSSGAVRGVCACASGRACSLLDKQENTLIPWCLPHTGNRHNHWAGLYGRLSWGGYFSTTVTDPEP : 1338
BtDNMT1a : SGSDWRDLPNIVVRLSDGTCTKKLEYTHDDKKVGKSSTGAYRGVCSCCMGKQCDPTDRQFNTLIPWCLPHTGNRHNHWAGLYGRLEWSGFFSTTITNPEP : 1221
BtDNMT1b : SGSDWRDLPNIAVRLSDGTYSKKLEYLYHDKKAGKSSIGAHRGVCSCCAGKMCDPIARQYNTLIPWCLPHTGRRHNHWAGLYGRLEWDGFFGTTITNPEP : 1361
CfDNMT1  : TGSDWRDLPNIAVRLSDGTFSKKLEYMHHDKRAGKSSTGALRGVCSCNTGRLCDPTDKQFNTLIPWCLPHTGNRHNHWSGLYGRLEWDGFFSTTVTNPEP : 1158
NvDNMT1a : TGSDWRDLPNIVVRLSDGTCTTKLEYKYNDKKAGVSSTGAPRGICSCSEGKSCSLRDKQVNTLIPWCLPHTANRHNHWAGLYGRIEWDGFFSTTITNPEP : 1317
NvDNMT1b : TGSDWRDLPNIVVRLSDGTCTTKLEYKYNDKKAGVSSTGAPRGICSCSEGKSCSLRDKQVNTLIPWCLPHTANRHNHWAGLYGRIEWDGFFSTTITNPEP : 1317
NvDNMT1c : VGSDWRNLPNIEMQLKDESYAKKLIYNYDDVEAGKSSNGELRGVCSCASGERCDPRDRQHGCLIPWHLVHSARKNKHWAGIYSRLQWDGFFNGAITNPQP : 1610
PxDNMT1  : PGSDWRDLPNISVTLSDGTKCKVLQYRHHDARSGRSPLGALRGVCACAAGGPCDPADKQENTLIPWCLPHTANRHNNWAGLY----------------- : 1242
TcDNMT1  : GGADWRNLPNVRVQLPDGTMTEVLQYRYRTKKQKDGE--PNRGVCACSVEKACDPNDRQSNTLIPWCLPHTADRHNNWAGVYGRLDWSGYFATTTNPEP : 1139
ZnDNMT1  : CGSDWRDLPNIVVRLSDGTFTKKLQYLHHDKKNGKSSTGGLRGVCGCANGKACDPMDRQYNTLIPWCLPHTGNRHNHWSGLYGRLEWDGFFSTTVTNPEP : 1122
            GsDWR1LPN6 6 Lsdg     L Y   dk  g ss ga RG6C C  g  Cdp d4Q nt6IPWcLpH3  4hnhWaG6Ygr  w g f tt t p p
```

```
AmDNMT1a : MGKQGRVLHPVQTRVVSVRECARSQGFPDSFRFYG--TILDKHRQIGNAVPPPLGVAIGHEIRNCIQNKDITEIDIKIQND------------------- : 1366
AmDNMT1b : MGKQGRVLHPEQTRVVSVRECARSQGFPDSFRFYG--NILDKHRQIGNAVPPPLGAALGFEIRKCLKND--NILKTKIEDLHD----------------- : 1444
ApDNMT1  : MGKQGRVLHPEQHRVVSVRECARSQGFKDSFIFHG--PIINKHRQIGNAVPPPMGTAIGHEIIKAIDKKPRYIYTPFS---------------------- :  602
BmDNMT1  : MGKQGRVLHPDQHRVVSVRECARSQGFPDTYLFAG--SVQDKHRQIGNAVPPPLGAALGREIKKALTLSLTTSCSS------------------------ : 1412
BtDNMT1a : MGKQGRVLHPVQSRVVSVRECARSQGFPDSFRFYG--TIFDKHRQIGNAVPPPLGEAVGHELRKCLRNESVKLIQSCN---------------------- : 1297
BtDNMT1b : MGKQGRVLHPEQTRVVSVRECARSQGFPDSFRFYG--NILDKHRQIGNAVPPPLGAALGFEIRKCLEDDDADHIKNKMEDFQD----------------- : 1442
CfDNMT1  : MGKQGRVLHPVQNRVVSVRECARSQGFPDSFRFYG--TILDKHRQVGNAVPPPLGAAIGQEIRKCIRDTTISSNTKFEFTKSCANTFMTRSNRSLDKNDK : 1256
NvDNMT1a : MGKQGRVLHPEQTRVVSVRECARSQGFPDSFRFYG--SIQDKHRQIGNAVPPPLAKAIGLEIRKSVSLKEANEVLDRVVVEEES---------------- : 1399
NvDNMT1b : MGKQGRVLHPEQTRVVSVRECARSQGFPDSFRFYG--SIQDKHRQIGNAVPPPLAKAIGLEIRKSVSLKEANEVLDRVVVEEES---------------- : 1399
NvDNMT1c : LGTQGPVLHPTQPRVVSVRECARAQGFPDEFVLPQKIGIHNMYRIIGRASSPFLGRAIGHEIARSLAARHRK---------------------------- : 1682
PxDNMT1  : ---------------------------------------------------------------------------------------------------- :    -
TcDNMT1  : MGKQGRVIHPDQNRLISVRECARSQGFPDKTKFFG--SVTSKYRQIGNAVPPPLAKAIGLEIKRALRNRHR----------------------------- : 1208
ZnDNMT1  : MGKQGRVLHPEQTRVVSVRECARSQGFPDTYRFFG--TILDKHRQIGNAVPPPMGAAIGFEIRKCIYAKEKTMRAESKMEIN------------------ : 1202
            gkqgrv hp q r  svrecarsqgfpd   f g      khrq gnavppp   a g e
```

Figure S1. Multiple sequence alignments of the deduced amino acid sequences of *AmDNMT1a*, *AmDNMT1b*, *ApDNMT1*, *BmDNMT1*, *BtDNMT1a*, *BtDNMT1b*, *CfDNMT1*, *NvDNMT1a*, *NvDNMT1b*, *NvDNMT1c*, *PxDNMT1*, *TcDNMT1* and *ZnDNMT1*. The numbering on the right represents the position of the last amino acid in that line. A black box indicates100% identity, and a grey box presents 100% similarity. Taxon abbreviations: *Am, A. mellifera*; *Ap*, *A. pisum*; *Bm*, *B. mori*; *Bt*, *B. terrestris*; *Cf*, *C. floridanus*; *Nv*, *N. vitripennis*; *Px, P. xylostella*; *Tc*, *T. castaneum*; *Zn*, *Z. nevadensis*.

```
TcDNMT2 : --------------MEILELYSGIGGMHWALKVSGVEGTIKAAVDINPTANSVYKHNFPHINLLNRNVQSLTPQFINKLGVNTILMSPPCQPFTRNGLQE :  86
NvDNMT2 : -------------------MSDSDD------DVPQLNPDTLAALNEFY-QEREEREKQ-FQAALEQNEN-QDATFDEDWQLSQFWYDEETISTLTQGAVQ :  72
DmDNMT2 : ------------MVFRVLELFSGIGGMHYAFNYAQLDGQIVAALDVNTVANAVYAHNYGSNLVKTRNIQSLSVKEVTKLQANMLLMSPPCQPHTRQGLQR :  88
CfDNMT2 : -------------------MYESDD------EEPQLSSSTLAALQEFLCKERFEQQNL-LELVSEEGQIPSNILFDENWQLSQFWYDDKTIEALVRGALN :  74
BtDNMT2 : --------------MRVLELYSGIGGMHYALQESGINGDVVAAVDINTVANSIYRYNFPNVLLMNCNIQSLSAKLINDLNIDTILMSPPCQPYTRVGLQR :  86
BmDNMT2 : --------MEEKMEHRILELYSGIGGMHCAWNESTIKGKVVAAIDINTVANDVYKYNFPETLLFTKNIQSLTPIEIEKYKIDTVLMSPPCQPFTRNGKNL :  92
AmDNMT2 : -------------MMRVLELYSGIGGMHYALQESGIKGDIVAAVDINTVANSIYKYNFPNVLLLNCNIQSLSAQEINNLNIDTILMSPPCQPYTRIGLQK :  87
AgDNMT2 : FPRRNTMESTKSEPHRVLELFSGIGGMRMALEQAGKEFEIVSAIDVNPIANEVYKHNFGAKTVRNGNILSLTAEKVTKLKVDTILMSPPCQPFTRNGKFN : 100
                           le6 sgiggm  a           aA6  n  an  y  n         n  sl             lmsppcqp tr G

TcDNMT2 : DINDERTKSFIHVLAILPDLKVTR-ILIENVKGFERSKMRDLLIETLEKCGFNYQEFILTPTQIGIPNTRHRYYCLAKKPPNVFNFKTGVLKTEFPN--- : 182
NvDNMT2 : STEGNAKIALISCPTLYKQLVSIAGERQVKILEFDKR---------FSIFGPDFIFYDYN-TPQDIPKDLYGQFDLVICDPPFLS--------------- : 147
DmDNMT2 : DTEDKRSDALTHLCGLIPECQELEYILMENVKGFESSQARNQFIESLERSGFHWREFILTPTQFNVPNTRYRYYCIARKG-ADFPFAGGKIWEEMPG--- : 184
CfDNMT2 : STPANGKIALISCPTLYSKLKKECDKRQITLFEYDSR---------FKIFGVDFIQYDYK-SPLNVPKCMSSQFDLVIADPPFLS--------------- : 149
BtDNMT2 : DISDNRSSSLLHVLSLIPQLRTLKHILLENVKGFENSEMRNAVLKCMNMSGFNYKELILSPCQFGIPNTRYRYYLLAKRKDSEFCFDHCILNFNLPEAVL : 186
BmDNMT2 : DENDPRTNSFLYFIDILDKLNTLQYILMENVKGFECSTVRNLFVEKLTYCGFVYQEFMLSPVSVGVPNSRLRYYCIAKRNNTWNFKRKDELITCLPK--- : 189
AmDNMT2 : DMLDNRSSSLLHVLSLIPQIKTLKYILLENVKGFEKSEMRNAVLKCINTSGFNYKELILSPCQFGIPNTRYRYYLLAKKNNLEFCFDQSVLNFELSDSIL : 187
AgDNMT2 : DINDRRSDPFLHICELLDKMPLVKFILMENVKGFENSQACEMYKARLREAGFHYQQYILSPHQFGVPNTRHRYYCIAKRHGADFKWKSE----------- : 189
          d  d r         6         il en6kg5e s             Gf 5    l p    6Pn r ry5 6a

TcDNMT2 : -------------------QQNAPHCFEISKVLEQNELTPYYLTDKVLTNYLETTDIRYSTSRNTCCFTKAYGRYVKGTGSVYSDLPEITP-EIFNQLSD : 262
NvDNMT2 : --------------------------------------------EECLTKTAITVKLLAKKQIVLC------------TGAVMSELAER-----LLNLKK : 186
DmDNMT2 : ------------------AIAQNQGLSQIAEIVEENVSPDFLVPDDVLTKRVLVMDIIHPAQSRSMCFTKGYTHYTEGTGSAYTPLSEDESHRIFELVKE : 266
CfDNMT2 : --------------------------------------------AECLTKTAVTIKFLMKKNIVLC------------TGAVMTELAER-----LLDVKR : 188
BtDNMT2 : KALPGSKHNLLLEG-----AKTDKNCYKLENILENVDKSQYLIPRKLLQKRAWLFDIRTSQSDGSCCFTKAYSHYAEGTGSVYSPYTEETIQQIFLEANK : 281
BmDNMT2 : -------------------TFAKPHC--LKDIIENNVPDDYLVPDKMLRK-ANIFDICYADSNRSCCFTKAYTHYVEGTGSVFTETSYDIVQKYLKLANY : 267
AmDNMT2 : KILPKSKYNLLTKKSCTQNSKTDKNCYTLENILEHVEGSQYLIPKKLLEKRVWLMDIRTSQSNGSCCFTKAYSHYVEGTGSVYCPYSEKTIKEAFSEANK : 287
AgDNMT2 : -------------------------------------------------------DIITTPQS--------------GCGAKQTLVG--------TIVDT : 212
                                                         l k     d         c           gtG v     e

TcDNMT2 : HEPGSSAYLK----------LAHGLKMRFFTPREVGRLMSFPEDFTFPENTSDKQKYMLLGNSINVRVVAELIKLLQ------ : 329
NvDNMT2 : CNFEPHHKNN------------LANEFWCYSNFDFDKYLT------------------------------------------- : 214
DmDNMT2 : IDTSNQDASKSEKILQQRLDLLHQVRLRYFTPREVARLMSFPENFEFPPETTNRQKYRLLGNSINVKVVGELIKLLTIK---- : 345
CfDNMT2 : CDFTPGHRNN------------LANEFCCFSNFNFDKMLE------------------------------------------- : 216
BtDNMT2 : YGKQSLEASE----------VLQKLMLRYFTPREVSRLMCFPEEFKFPEHITCKQRYRLLGNSINVYVVSRLIFLLYTEKKIT : 354
BmDNMT2 : FEVGSDEFLQ----------TLKKLKLRFFTSKEILQLMSFPSEYSFPKTVTRKQCYRLLGNSVNVKVISELLQILFDE---- : 336
AmDNMT2 : YERQSLEVSK----------ILEKLMLRYFTPREICRLMCFPEEFIFPEYITDKQKYRLLGNSINVYVVSRLIFLLYTERKIT : 360
AgDNMT2 : QQDALEQYG-----------LKSATLLKHLPLMDVCT---------------------------------------------- : 238
```

Figure S2. Multiple sequence alignments of the deduced amino acid sequences of *AmDNMT2*, *AgDNMT2*, *BmDNMT2*, *BtDNMT2*, *CfDNMT2*, *DmDNMT2*, *NvDNMT2*, and *TcDNMT2*. The numbering on the right represents the position of the last amino acid in that line. A black box indicates100% identity, and a grey box presents 100% similarity. Taxon abbreviations: *Am, A. mellifera; Ag, A. gambiae; Bm, B. mori; Bt, B. terrestris; Cf, C. floridanus; Dm, D. melanogaster; Nv, N. vitripennis; Tc, T. castaneum*.

```
ZnDNMT3 : ---------------------------------------------------------------------------------------------------- :    -
NvDNMT3 : TPGKLVWGYFRSGWWPDLNIVT-------------------------ALIIRAEDAGMIPSSEKIWVSWIGESRISELNAK-CIDKFSNHLERRLDNLAT :  395
CfDNMT3 : ---------------------------------------------------------------------------------------------------- :    -
BtDNMT3 : NNELRNKGSRWGEEYHEVRRVTRGSIKISKDLFVGKLVWGCCSGWWPALIIDADHVGMLSEAGKSWVYWIGEARISLLSEKTQIEPFSYNLKSRLTQNSN :  500
AmDNMT3 : ---------------------------------------------------------MLSEEGKLWVYWIGEARISLLNEKTQIEPFSCNLKARLTQNLN :   43

ZnDNMT3 : ----------------------------------------------------------------------MSSLESRRRKKIVAKRLLNPSD-------- :   22
NvDNMT3 : NSKTKVTCKKQKDEACFKTIQLLKKHFTGGALVKPYIAWIKNNILP-YKNKLDELHFYPYPESLSDKLNNLKIVNSEKNEKYLRQQEKERLC----KAFE :  490
CfDNMT3 : ---------------------------------------MKDNL---FCVTVDDLIFYPYPKNIQERLDTLREKNIKATERFISNQRSSPETSPTKKQAV :   58
BtDNMT3 : EARMRVID---------ATMQMLRRLLGG-TLTKPYFTWIENNL-QCAIETLDEIKFYPYPDKIQQRLNGLREKNAKITKKYLLDQKRESQG----KKLA :  585
AmDNMT3 : VPRIRAID---------ATMQMLRKKLGG-TLTKPYFTWIESNFPKNMIEMLDEIKFYPYPVKMQQRLDHLREKNAKVTERYLLDQKRENQE----KKLA :  129
                                                    n         d   fypyp      l  6   n     4 6  q          k

ZnDNMT3 : ---------------------PFEGPEGSLVWGKLRNFPWWPGIIVDEHACGLSCIPSGQFCIFWFADRRVSGVQNKWMKDFKKHYEQCFDPT--VKQAL :   99
NvDNMT3 : PKNLIKKIEKFEKPIEKGGINIVDQKYGMIVWAKMQGYSWWPCVIMDYQHLNRKQPHVAHQWVMWYGDYKYSQVQYRQILTFPTGMDRMESKITATKDEL :  590
CfDNMT3 : EK--CKDTNTSWKQIEDERLPLQNQNPGVIAWAKIAGHCWWPAMIIDYRDCCLKEPSFGCQWIMWYGDYKVSEVRHLEFLKFYKGLEKMRDYIQNTVKQC :  156
BtDNMT3 : EK--AKDS-LQRGNVDLTHLPLKEQNPGIITWAKIAGHNWWPAMIIDYRDCCMREPSFGCQWIMWYGDYQLSEVHHQLFMRFDKGMEKMRDYINNTKKHI :  682
AmDNMT3 : EK--SKDS-PQKVNVDLTLLPLKEQKPGIIAWAKIAGHNWWPAMIIDYRDCCMREPTFGCQWIMWYGDYKLSEVHHQLFLRFDKGMEKMRDYTSNTKKHI :  226
           k   k                  q  G 6 WaK6 g  WWP 6I6Dy  c    p  g qw6mW5gDy  S V       F kg e m d    tk

ZnDNMT3 : YVSGVEEAVKLCAKRLGHNAESWTKKEALCWAQAGFQRSANRKVEEVTPIFVPDGVQDDLLLIKRCRKSNEKNNKLLENCGSSSEASSSQPSFQYDDLKK :  199
NvDNMT3 : FCKAVLQAAKDYCDKLGYLTEPWKIKDVIHLFYK------SKDIYKLKNAELTEPNEE----DLYSQTIKKQLRKQINNQPISEERKKKIL--ECKNLNL :  678
CfDNMT3 : YLDGVLQASKDYCSRLGCSTDNWTLDNVFEYFSN------MNNIHVPYNQ--LQVSDSNKIYDKYSDEIVKKINEFKSKPNVDAERKNDIK--TSDALHR :  246
BtDNMT3 : YLFGVLQASKDYCSRLGFKTENWTVNDALKYFAR------KEDSKRSCAQ---RKEDSVKIYDKYSACIAKKLNELKNNANVDDGRTNDIK--NSDDLRS :  771
AmDNMT3 : YLVGVLQASKDYCSRLGFDTSNWTLDDAFEYFSK------PNHYDYASSANTWRREDSVKIYDKYSARIAEKLNELKDNPNVDDQRANDIN--NSDDLRS :  318
          5  gVl2A Kdyc 4LG  t  Wt       f                              dkys  i  k n    n      r   i     d L

ZnDNMT3 : VKKGEMDIEKLCLRCRSSKVQIVGEHPYFKGSLCKICMETVKEHIFTFGADGIHMHCAICSNAGEVFVCSNPQCCKVYCHVCIDLLASEGAKNWIREKEP :  299
NvDNMT3 : LLSGKLPLESLCISCLESGE-ELEDHPFFHASMCEKCLEDFSPKIFAYGNDAKCFYCTLCGGDDLVAVCDSMSCPRVFCTACIKYIICPEFYEDILLKHP :  777
CfDNMT3 : VISGECTVEKLCLKCLRFSKDKMEEHPFFIGSLCKECSYAFKPCMFVHGNDGKCFYCTVCAATGTVLICDTDDCPRVYCTACLKFLICPKAYDDMLSEDP :  346
BtDNMT3 : AMNGNIAFESLCLKCLRVAEGKTEIHPFFEGSLCKSCSDHYKPCMFLFGNDAKCFHCTVCAASGMMIICDKEDCPRVYCTACMKHLLCPTTYEQVLQEDP :  871
AmDNMT3 : AIKGEISFDSLCLKCLRVSNDEMDIHPFFEGSLCKDCSERYKPCMFVFGNDSKCFYCTVCAASGMVIICDKEDCPRVYCTACMKHLLCPTTYEQVLQEDP :  418
             G    e LC6 Cl         HP5F gS6Ck C    kp 6F  GnD kcf Ct6C   g 6 6Cd   Cp4V5CtaC6k 6 cp  y  6l   P

ZnDNMT3 : WLCFLCDDPLS---SMHGFIKPRPDWMNCQRISALFPANNIMSS----IPLSKKSKSGLRVLSLFDGISTGMVVLKMMGIKVEKYYASEVDKDAINVSKV :  392
NvDNMT3 : WYCFLCDPSS--ISTNNVVIKIRNDWR--YKMISLYRINCDEEAPSNLERLDR-NRK-IRVLSLFDGIGTGLVVLKHLNVNIECYYASEIDPDSMQVSFF :  871
CfDNMT3 : WKCFLCRDESK--QPANMLLHPRLDWK--EKVSTMFRTASNPASKDINFENYKNQKKPIRVLSLFDGLSTGFLVLLNLGIVVDVYYASEIDKNALTISSA :  442
BtDNMT3 : WECFLCATNNKPRPSSDSIIKPRANWK--DKIINMFRTNCKSNPQELM-KHNWEKKK-IRVLSLFDGLGTGLLVLLKLGLTVDTYYASEIDPDALMVTAA :  967
AmDNMT3 : WECFLCKSRS---FTTDTIVRPRANWK--DKIINMFRTSCDSNVEHLVAKHNSEKRK-IRVLSLFDGLGTGLLVLLKLGFIVDAYYASEIDQDALMVTAS :  512
          W CFLC             6 pR 1W    46  65r                  4k 6RVLSLFDG6 TG 6VL  6g  6  YYASE6D 1a6 63

ZnDNMT3 : NHGDSIEHIGDVELLSPEKLSRLGPIDLLIGGSPCTELSLVNPARKGLYDTEGSGYLFFDFYRVLMTLQVLHG-KNIFWIFENTASMPYRLRNVITRFLG :  491
NvDNMT3 : NHGNEIIQLGDVRNIDEKKIKEIAPIDLLIGGSPCNELSLANPKRRGLDDPEGTGILFYDYVRIMKLVKKHNKKRHLFWLFENVASMPKKFRNQISKNLG :  971
CfDNMT3 : HFGDRITYLGDVRDITKEKIQEIAPIDLLIGGSPCNDLSLVNPARLGLHDPKGTGILFFEYCRIKKLMKKANKDRHLFWLYENVASMPSEYRLEINKHLG :  542
BtDNMT3 : HFGDRIVHLGNVKDITKEKIQEIAPIDLLIGGSPCNDLSLANPMRLGLYDPKGTGILFFEYCRIKDLLKKVNNACHLFWLYENVASMPTEYRLEINRHLG : 1067
AmDNMT3 : HFGDRILQLGNVKDITCNTIKEIAPIDLLIGGSPCNDLSLANPARLGLHDPRGTGVLFFEYRRILKLVRKLNNERHLFWLYENVASMPSEYRLEINKHLG :  612
            G1 I  6G1V  6   k6 e6aPIDLLIGGSPCn LSL NP R GL Dp G3G LF5 5 R6  l6 k n   h6FW65ENvASMP   R  I 4 LG

ZnDNMT3 : CDPVVIDASWLSAQRRARFFWGNIPGLGRTELPEINMKLDECLMPGMERKAVVEKIRTVTTQSNSLLQG--KNRIFPVKMKEEMDAVWITELEVIFGLPL :  589
NvDNMT3 : REPKFLDSADFSAQHRPRLYWGNLPWGPYQVN---NVVLQDVLRKRCNRQALVKKIMTVTTRTNSLNQT--KENLKPVLMDGKKDMLWVTELEKIFGFPM : 1066
CfDNMT3 : REPDVIDAADFSPQHRLRLYWHNFPFNPYMPLFQNQQDVQDKLTPNLNRKALCKKLRTVTCRTGSLLQG--KAEVKPIMMKGESDRLWITELEEIFGFPR :  640
BtDNMT3 : QEPDTIDSADFSPQHRLRLYWHNLPIEIHSLSTQREQDVQDILTPHCQRYSLVKKIRTVTTKVNSLKQG--KLALKPILMKDESDSLWITELEEIFGFPR : 1165
AmDNMT3 : QEPDVIDSADFSPQHRLRLYWHNFPIEPRLLSSQREQDVQDILTPHCQRYSLVKKIRTVTTKVNSLKQGDGKLALKPILMKDESDSLWITELEEIFGFPR :  712
           eP  6D adfS QhR Rl5W N P             6qd L p   R  6vkK6rTVTt  nSL Qg  K  6kP6 Mk e D 6W6TELE IFGfP

ZnDNMT3 : HYTDTGNLQLRERRQLLGRAWSVPVVKHILQPLKLYFKCEREKSVKQGKMTT------------ :  641
NvDNMT3 : HYTDTN-LQKTRRLQLIGKAWSVQTLTAILRPVFLF---------------------------- : 1101
CfDNMT3 : HFTDVKNLSATNRQKLLGKSWSVQTLTAILRPLCFYFKCKEDETSNNISALHKDISLLYRGKQF :  704
BtDNMT3 : HYTDVKNLSASKRQRLIGKSWSVQTLTAIFRSLCPFFECNTIETN------------------- : 1210
AmDNMT3 : HYTDVKNLSATKRQRLIGKSWSVQTLTAIFESLCPFFERDIVEIEG------------------ :  758
          H5TD  nL    R  L6G4 WSVqt6taI   6  5f
```

Figure S3. Multiple sequence alignments of the deduced amino acid sequences of *AmDNMT3*, *BtDNMT3*, *CfDNMT3*, *NvDNMT3* and *ZnDNMT3*. The numbering on the right represents the position of the last amino acid in that line. A black box indicates100% identity, and a grey box presents 100% similarity. Taxon abbreviations: *Am, A. mellifera; Bt, B. terrestris; Cf, C. floridanus; Nv, N. vitripennis; Zn, Z. nevadensis*.

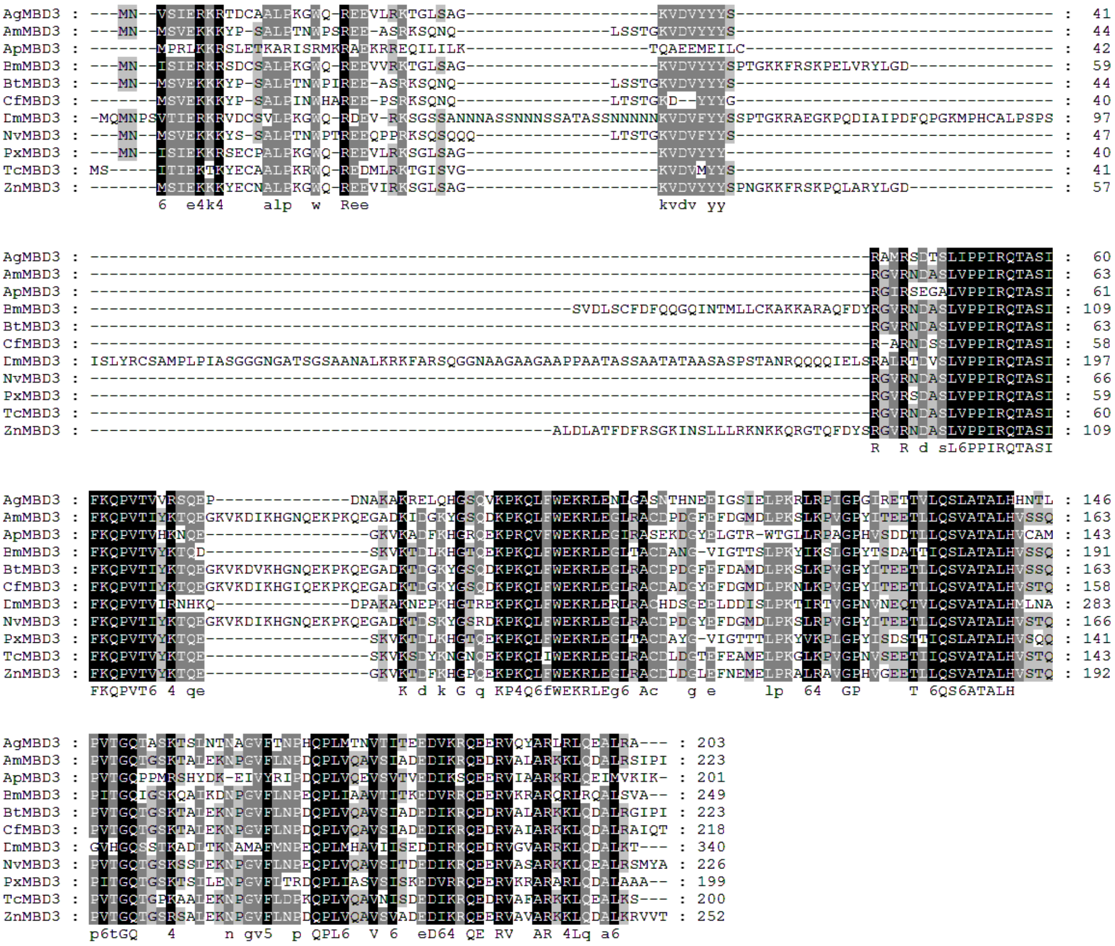

Figure S4. Multiple sequence alignments of the deduced amino acid sequences of *AmMBD3*, *ApMBD3, AgMBD3*, *BmMBD3*, *BtMBD3*, *CfMBD3*, *DmMBD3*, *NvMBD3*, *PxMBD3*, *TcMBD3* and *ZnMBD3*. The numbering on the right represents the position of the last amino acid in that line. A black box indicates100% identity, and a grey box presents 100% similarity. Taxon abbreviations: *Am, A. mellifera; Ap, A. pisum; Ag, A. gambiae; Bm, B. mori; Bt, B. terrestris; Cf, C. floridanus; Dm, D. melanogaster; Nv, N. vitripennis; Px, P. xylostella; Tc, T. castaneum; Zn, Z. nevadensis*.

```
AmTET2 : VPSSTATSRSDDCASKAIKSEPDQKSQEGNNYKFRGDGGPAKVSPGVGSWCCRRGGTEQPTPEHLREGCCQ--GLQTRDEILADG---TDKSD-VKNEGP : 1089
BmTET2 : YYIEQIKSEHSPPGHKIYKNMNYS-PPRDEPYLFPGDGGPTAIKNEVGYSCCRQGSTKKPPPEHLRDGACP--GYQTKDEVLEDEPETTDKNDPKN-QKA : 1380
BtTET2 : VPSPTVSSKSDGCASKEIKSEPDQRAQESNNYKFRGDGGPAKVSPGVGSWCCRRGGTEQPTPEHLREGCCQ--GLQTRDEILADS---AEKSD-VKNEGT : 1512
PxTET2 : YYVEQIKSEHSPPGHKIYK-SSFGHLSKSEPYLFRGDGGPVIIRNEVGYSCCRQGSTKKPPPEHLRDGACP--GLQTKDEVIEDDPDATDKNDPKCQGKP :  781
NvTET2 : LSVRSEEQEVEERMHVLATLLSPLYLSLAPEAFNNQTQFEREASECRLGFKPGRPFSGVTACIDFCAHAHRDLHNMNNGCTVVVSLTKHRSFSKPDDEQL : 1800
TcTET2 : TSVS-SPSNYGNSGNAQPGYPQVNSSPQLASHSSSGYPGSGQFSG-TSQEMWQEESVWQEEAKPEQNDRVN-LNTRIKTMILSKQQENSEEKKVENTTGH :  771
ApTET2 : -----LPFGIDPRVTQ-----------KNGYVFCGNGGPNPIDVVAGPWCCRMGGTDTPTTKHLSDGCCH--GLKTLDEG-------IDPVEMKQENGL :  219
AgTET2 : PPPTKKESKSKSKKSSAASTASSGASTANSVASPGPGGSGSTGAGGGSGGSGSGSPRAHTPGSQKSTGSGKGGSGSNGGSSNAALDLVTGVLTEKHSSPL : 1800
DmTET2 : ALGHGAPPHLSHHGMLEPKIEDMGMLGHGGGYAYLGSEGKPLNN---GFSCCRQGGTRPPTAEHLKDGTCLGLGIQPKEELIDEDELIDTHGNGLKPIGG : 1594
ZnTET2 : NSNCNSQNDVRTATPPDKVTESTKVKTEQLSYLFAGDGGPLPLEKTKGSWCCRQGGIETPTPEHLRDGCCQ--GFQTADEQDTKQPWSPQQPDASTGDGC :  688
CfTET2 : ----DTSSIAENEESRKIVP----------DYKFRGDGGPAKVSPETGSWCCRRGGTKQPSPEHLKDGCCQ--GLQTKDEMLADS---PQAAE-LKNEGP :  402
                                            g  g                            g

AmTET2 : QSPR------------NGGAPSTTKLQDHLEKLKNNVRSEVPDCNCFPADKCPPEPGSYYTHLGAAASLPDLRNDLERRTGLKGNAIRFEKVIYTGKEGK : 1177
BmTET2 : TAPTPD-----VTKPKENQFNYSKEYLENLERLRTNSRTEVPDCNCFPADKNPPEPGSYYTHLGSASSLAELRELEARTGLSGKELRIEKICYTGKEGK : 1475
BtTET2 : QSPR------------TGSVPSTTKLQDHLDKLKNNVRSEVPDCNCFPADKCPPEPGSYYTHLGAAASLPDLRNDLERRTGLKGNAIRFEKVIYTGKEGK : 1600
PxTET2 : PTPGPDA-LAKVTKEVPNQFNYSKEDLENLEKLKNNSRTEVPDCNCFPTDKNPPEPGSYYTHLGAASTLAELRELESRTGLSGKELRIEKVCYTGKEGK :  880
NvTET2 : HVLPLYIMDDSDEFGSKEGQEAKIKSGAIEVLTKYPCEVRVRSVPLQPCRRHGKKRKEDEPDVASNKKDTKSPAEKFVDTNRPQMYQDPSRPPLRDSQLS : 1900
TcTET2 : FLWYSHHHRFIVNGGGGAKLTTTKPKQLPKKRYSSPYENEIPYCNCVRAGRGAAEPGTFYTHLGCANNLINLRHDLETRTGVKGRAIRIEKIRYTGKEGK :  871
ApTET2 : NNSQ-----------------CSDNLDD---KQKTNIKATIPDCNCFPTDQAPPEPGPFYTHLGSAYSLIELRTNMENMSGIRGNGIRMEKVLYTGKEGK :  299
AgTET2 : HGQGAGGAGGALVAAGGGNGGTGGSSAFTAPNTINSPNSLANNGANNTNSTLIDMASMIDNFTDAQLQSNQISSTVLDSPYSYDYNTGQYIDNRQYYNNW : 1900
DmTET2 : VGKAKGKQKPDEIPEIVVKHEKINPMFDTTDRLEKGNKTEIPECECFQSDKNPPEPGTYYTHLGTASSLMDLREFEERCNLTGRQLRIEKIVYTGKEGK : 1694
ZnTET2 : HNTN-K----------GFGGMSAKEFQDHLERLKNNVRAEVPDCNCFPPDKCPPEPGSYYTHLGASANLTDLRKDVEKRMGFKGKALRIEKVMYTGKEGK :  777
CfTET2 : HSPRTP----------ASAATTTTKLQDHLDKLKNNVRTEVPDCNCFPADKCPPEPGSYYTHLGAAASLPDLRNDLERRTGLKGDAIRFEKVIYTGKEGK :  492
                                                  p c c        epg  yth g    l   r   e      g   r e   ytgk gk

AmTET2 : TTQGCPMAKWILRRSGLEEKILTIVKHRQGHKCPTAWIVVAMVAWEGVPTHEADRIYSLLSHKLNRFGLPTTRRCGTNEPRTCACQGLDPETCGASFSFG : 1277
BmTET2 : TAQGCPMAKWVIRRANYTEKVLVVVKCRNGHKCPTAWIVVCLVAWEGIPQSEADLDYTLLSHKLNRYGLPTTRRCATNENRTCACQGLDPETCGASYSFG : 1575
BtTET2 : TTQGCPMAKWILRRSGLEEKILTIVKHRQGHKCPTAWIVVAMVAWEGVPTHEADRIYSLLSHKLNRFGLPTTRRCGTNEPRTCACQGLDPDTCGASFSFG : 1700
PxTET2 : TAQGCPMAKWVIRRTNYTEKVLVVVKCRNGHKCATTWIVVCLVAWEGIPQSEAALDYTLLSHKLNRYGLPTTRRCATNENRTCACQGLDPETCGASYSFG :  980
NvTET2 : LEMTSMLEGMDAQLQSSQVSSTVLDSPVSMYQSWNYQTEQQWSRPGWLDQRKSNWMNPWTEYGPFGGLDREVKVDPDSTSLDDSRPRSAVSQEDYRPASR : 2000
TcTET2 : TAQGCPIAKWVIRRSGSDEKYLIIVKHRPGHSCPSAFIVVCIVMWDGLPQPTSDELYTLLTSKLNKFGLANRRRCATNESKTCACQGLNPDTCGASFSFG :  971
ApTET2 : TTQGCPLAKWVIRRSSTDEKLLVVVKNRRGHKCQHSWIVICIVSWEGILSDEADFLYTMLSHKLNKYGVPTTRRCGTNDPRTCACQGLDPDTCGASFSFG :  399
AgTET2 : PTDYYGNRGDPSKLAGLGRNEEIPDTTTRPGSNSSNSAFSPSIPDPTKTPTPIHMDDYGSTKLGQGQVHHHHHQLQQQQQQQQQQHQQQHQQHQQQHQQQ : 2000
DmTET2 : TSQGCPVAKWVIRRADLEEKILVVVKKRPGHRCIAAYIVVCMVAWDGMPRLEADNAYKNLIPKLNKYGLPTTRRCATNENRTCACQGLDPESSGASYSFG : 1794
ZnTET2 : TTQGCPLAKWIIRRSSMEEKILCIVKHRQGHKCPTAWIVVVMVAWEGVPSHEADRVYNLLTHKLNRYGLPTTRRCATNEPRTCACQGLDPDTCGASFSFG :  877
CfTET2 : TTQGCPMAKWIIRRSGMDEKILTIVKHRQGHKCATAWIVVAMVAWEGVPTHEADRIYSLLTHKLNRFGLPTTRRCGTNEPRTCACQGLDPDNCGASFSFG :  592
         t qgcp akw   r    ek l  vk r gh c    iv     w g         y  l  kln  g     rc tn   tcacqgl p   gas sfg

AmTET2 : CSWSMYYNGCKYARSKTVRKFRLSVRSEEQEVEERMHVLATLLSPLYLSLAPEAFNNQTQFEREASECRLGFKPGRPFSGVTACIDFCAHSHRDLHNMNN : 1377
BmTET2 : CSWSMYYNGCKYARSKTVRKFRLSVKTEESEIEERIHVLATLLSPLYMNLAPRAFDNQCQFEKEASDCRLGFKPGRPFSGVTACIDFCAHAHRDLHNMNN : 1675
BtTET2 : CSWSMYYNGCKYARSKTVRKFRLSVRSEEQEVEERMHVLATLLSPLYLSLAPEAFNNQTQFEREASECRLGFKPGRPFSGVTACIDFCAHSHRDLHNMNN : 1800
PxTET2 : CSWSMYYNGCKYARSKTVRKFRLSVKNEESEIEERMHVLATLLSPLYMNLAPKSFENQCQFEKEASDCRLGFKPGRPFSGVTACIDFCAHAHRDLHNMNN : 1080
NvTET2 : QNSNSESPRANYTQSSRGHPISPRASLPSTPVESIYSTPPRTCPSTPHEPHDQRLISPRSSGYGSAPSEFSLSSPSPRNYQNSLDGRQNSISPRAAYQNS : 2100
TcTET2 : CSWSMYYNGCKFSRSKFVRKFRLNVQPEEKIVEEKLQILATYLSPIYRSLAPVAFRNQCFFEEGGRECRLGLRPGRPFSGVTACLDFCAHSHKDSHNMVN : 1071
ApTET2 : CSWSMYYNGCKYARSKDVRKFRLSVRTEEQELEERLHVLATNLSPLYKSLAPRSYNNQIQCEREGSDCRLGLKPGRPFASVTACIDFCAHAHRDFHNMHN :  499
AgTET2 : QQHQQQQSQQQTQNTYHTLQQPEQGFVKPKPPDYNPAAGYGYHHPNTPMGYGYHHHHHPPYHHTPYDPYQNYNYGYPPQSYHHHHHTTYHNMYPAAAAAA : 2100
DmTET2 : CSWSMYYNGCKYARSKTVRKFRLSVKSEEAAIEDHMNLIATLLAPVFKQVCPRSYDNQTKYEHEASDCRLGLEPGKPFSGVTACLDFCAHSHRDLHNMQD : 1894
ZnTET2 : CSWSMYYNGCKYARSKTVRKFRLSVRSEEQEVEERMHVLATLLSPLYHSLAPDAFKNQTQFEREASECRLGFKPGRPFSGVTACIDFCAHSHRDLHNMNN :  977
CfTET2 : CSWSMYYNGCKYARSKTVRKFRLSVRSEEQEVEERMHVLATLLSPLYLSLAPEAFNNQTQFEREASECRLGFKPGRPFSGVTACIDFCAHSHRDLHNMNN :  692
         cswsmyyngck  r3k vrkfrl v  ee   e      at l p      p    nq   e     crlg  pg Pf  vtac dfcah h d hnm

AmTET2 : GCTVVVTMTK--HRTLSKPEDEQLHVLPLYIMDTTDEYGSKEGQDEKVRAGAVEVLTKYPCEVRVRSVPLQPCRRHGKKRKEDEPDTISSKKDSSKNVNE : 1475
BmTET2 : GCTAVVTLAK--HRALSRAGDEQLHVLPLYVLDDTDEFGSKEGQDEKVASGALEILDKYPMEVRVRSVPLQPCRRHGKKRKEEDSGDSANNSTNNTPQQS : 1773
BtTET2 : GCTVVVTMTK--HRSLSKPEEEQLHVLPLYIMDTTDENGSKEGQDEKVRAGAVEVLTKYPCEVRVRSVPLQPCRRHGKKRKEDEPDTISNKKDSSKSTAE : 1898
PxTET2 : GCTAVVTLAK--HRNLTKPPDEQLHVLPLYVLDNTDEFGSKEGQDEKIKNGALEILDKYQMEVRVRSVPLQPCRRHGKKRKDEPEADANTSNSSNNSQQS : 1178
NvTET2 : SYNLESPQRNTPSRNNQHTSTPIGSEAARRNQSPSMNQLRSPSLNSSDSYSSNISSPRYPVDHNQRFPGSMAQNIAEMQDSKSTPKAPISQIPQQSESNS : 2200
TcTET2 : GCTVVVTLTK--HRKGEKPEDEQLHVLPLYVVEGTDEFDSQGGQEEKIRMGSIEVLTKFETEIRS----------------------------------- : 1134
ApTET2 : GCTVVVTLNK--HRGFQKPDDEQLHVLPLYVVDESDEFGDKQAQSDKFKNGSVEMLSKFPCEVRVRSVPLTPCRR--KKRLAEEPESP------------ :  583
AgTET2 : AAAQTQLPGTGPAPPPPPTAVIPAPPPPSNWNLYPPHLGTAPSTAQLHVAAAAAGALGKQSELGGAVGAGMMSQSAVVPQAPAAPPSAPLPPKETLGEVT : 2200
DmTET2 : GCTVHVALLKPGNRDTRLPDDEQFHVLPLYTMDGTDEFESVEGQRDKHRTGAVQMLDKFPCEVRVRSTPLIPCRRHGKKRKDGDEAAPPDGDQDAVGDAN : 1994
ZnTET2 : GCTVVVSLTK--HRTLSKPDDEQLHVLPLYVMDDTDEFGSKEGQDAKVKSGAVETLTKFPCEVRVRAVPLQPCRRHGKKRKEDEPDAVIGRKDPATVHRS : 1075
CfTET2 : GCTVVVSLTK--HRALSKPEDEQLHVLPLYIMDDTDEFGSKEGQEKKVRSGALEILSKYPCEVRVRSVPLQPCRRHGKKRKEDESDISNGKKDTVKTFSE :  790
         gct  v   k   r       eq hvlply     de      q  k   g    l     e r r
```

Figure S5. Multiple sequence alignments of the deduced amino acid sequences of *AmTET2*, *ApTET2*, *AgTET2*, *BmTET2*, *BtTET2*, *CfTET2*, *DmTET2*, *NvTET2*, *PxTET2*, *TcTET2* and *ZnTET2*. The numbering on the right represents the position of the last amino acid in that line. A black box indicates100% identity, and a grey box presents 100% similarity. Taxon abbreviations: *Am, A. mellifera; Ap, A. pisum; Ag, A. gambiae; Bm, B. mori; Bt, B. terrestris; Cf, C. floridanus; Dm, D. melanogaster; Nv, N. vitripennis; Px, P. xylostella; Tc, T. castaneum; Zn, Z. nevadensis*.

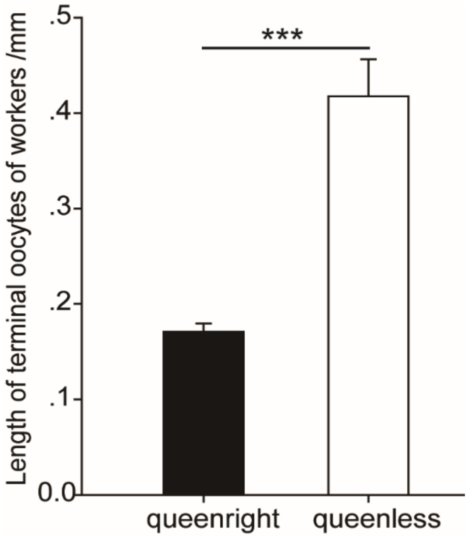

Figure S6. Length of the terminal oocytes of workers in queenright and queenless colonies. Data in the figure represent mean ± SM of four biological replicates, and the asterisks indicate significant difference between two treatments by independent sample T-test ($P < 0.001$).

Table S1. Primers used for qRT-PCR.

Primer name	Primer sequence（5'→3'）
DNMT1a F	CATATTCCGACTGCGAGTG
DNMT1a R	ACGCCACGATGTGCTCC
DNMT1b F	GTGGGTTATCCGAAGGT
DNMT1b R	TGTTGCATTAGGGTTGT
DNMT2 F	ATAGTGGTATTGGTGGAA
DNMT2 R	TAAGGTTGACATGGAGG
DNMT3 F	ACTATAAGCCCTGCATGTTCC
DNMT3 R	ATCATGCCAGAGGCTGCG
TET2 F	CACTCCGCTACCTTACATAC
TET2 R	GCACGGTTCTTGCTCAT
MBD3 F	CCGATTAGGGAAGAAGC
MBD3 R	CGTATTTGCCATCAGTTTT
EF1a F	CGTTTACCGCTTCAGGACGT
EF1a R	GCATGCCTGGTTTCAGAATACC

Table S2. Sequences analysis of genes involved in DNA methylation in *Bombus terrestris*.

Gene	Open Reading Frame	Number of amino acid	Relative molecular weight of protein	Isoelectric point of protein
DNMT1a	4,329 bp	1,442 aa	163.62 KDa	6.56 pH
DNMT1b	3,894 bp	1,297 aa	148.48 KDa	8.42 pH
DNMT2	1,065 bp	354 aa	40.73 KDa	8.82 pH
DNMT3	3,633 bp	1,210 aa	138.47 KDa	7.87 pH
TET2	8,268 bp	2,755 aa	306.77 KDa	6.93 pH
MBD3	672 bp	223 aa	24.87 KDa	9.21 pH

Table S3. Gene names, species names and GenBank accession numbers used for the phylogenic analysis.

Gene	Species	GenBank accession number
DNMT1a	*Bombus terrestris*	XP_008181290.1
DNMT1b	*Bombus terrestris*	XP_012167869.1
DNMT1a	*Apis mellifera*	NP_001164522.1
DNMT1b	*Apis mellifera*	XP_006562865.1
DNMT1a	*Nasonia vitripennis*	NP_001164521.1
DNMT1b	*Nasonia vitripennis*	XP_008217946.1
DNMT1c	*Nasonia vitripennis*	XP_001607336.1
DNMT1	*Camponotus floridanus*	XP_011257755.1
DNMT1	*Zootermopsis nevadensis*	XP_021941799.1
DNMT1	*Bombyx mori*	BAP86925.1
DNMT1	*Plutella xylostella*	XP_011556728.1
DNMT1	*Acyrthosiphon pisum*	XP_008181290.1
DNMT1	*Tribolium castaneum*	KYB29654.1
DNMT2	*Bombus terrestris*	XP_003400893.1
DNMT2	*Apis mellifera*	XP_006563008.1
DNMT2	*Nasonia vitripennis*	NP_001129999.1
DNMT2	*Camponotus floridanus*	EFN60586.1
DNMT2	*Bombyx mori*	NP_001036934.1
DNMT2	*Tribolium castaneum*	EFA09160.1
DNMT2	*Drosophila melanogaster*	NP_477475.2
DNMT2	*Anopheles gambiae*	XP_306830.2
DNMT3	*Bombus terrestris*	XP_003393693.2
DNMT3	*Apis mellifera*	NP_001177350.1
DNMT3	*Nasonia vitripennis*	XP_008204446.1
DNMT3	*Camponotus floridanus*	EFN74862.1
DNMT3	*Zootermopsis nevadensis*	XP_021915979.1
MBD3	*Bombus terrestris*	XP_003396187.1
MBD3	*Apis mellifera*	XP_392422.2
MBD3	*Nasonia vitripennis*	NP_001164526.1
MBD3	*Camponotus floridanus*	EFN64427.1
MBD3	*Zootermopsis nevadensis*	XP_021933495.1
MBD3	*Bombyx mori*	XP_004929675.1
MBD3	*Plutella xylostella*	XP_011549226.1
MBD3	*Acyrthosiphon pisum*	NP_001156167.1
MBD3	*Tribolium castaneum*	XP_969537.1
MBD3	*Drosophila melanogaster*	NP_001262421.1
MBD3	*Anopheles gambiae*	XP_318432.4
TET2	*Bombus terrestris*	XP_012169992.1
TET2	*Apis mellifera*	XP_016770977.1
TET2	*Nasonia vitripennis*	XP_008203340.1

TET2	*Camponotus floridanus*	EFN73124.1
TET2	*Zootermopsis nevadensis*	KDR09818.1
TET2	*Bombyx mori*	XP_012544637.1
TET2	*Plutella xylostella*	XP_011554443.1
TET2	*Acyrthosiphon pisum*	XP_008183450.1
TET2	*Tribolium castaneum*	XP_008193024.1
TET2	*Drosophila melanogaster*	NP_001246581.1
TET2	*Anopheles gambiae*	XP_001688023.1
